# Neurodegeneration and Astrogliosis in the Human CA1 Hippocampal Subfield Are Related to hsp90ab1 and bag3 in Alzheimer’s Disease

**DOI:** 10.3390/ijms23010165

**Published:** 2021-12-23

**Authors:** Melania Gonzalez-Rodriguez, Sandra Villar-Conde, Veronica Astillero-Lopez, Patricia Villanueva-Anguita, Isabel Ubeda-Banon, Alicia Flores-Cuadrado, Alino Martinez-Marcos, Daniel Saiz-Sanchez

**Affiliations:** CRIB, Neuroplasticity and Neurodegeneration Laboratory, Ciudad Real Medical School, University of Castilla-La Mancha, 13071 Ciudad Real, Spain; melania.gonzalez@uclm.es (M.G.-R.); sandra.villar@uclm.es (S.V.-C.); veronica.astillero@uclm.es (V.A.-L.); patricia.villanueva@uclm.es (P.V.-A.); Isabel.Ubeda@uclm.es (I.U.-B.); alicia.flores@uclm.es (A.F.-C.)

**Keywords:** amyloid-β, tau, GFAP, autophagy, cavalieri, optical fractionator, SWATH-MS

## Abstract

Alzheimer’s disease (AD), the most prevalent neurodegenerative disorder, is characterized by executive dysfunction and memory impairment mediated by the accumulation of extracellular amyloid-β peptide (Aβ) and intracellular hyperphosphorylated tau protein. The hippocampus (HIPP) is essential for memory formation and is involved in early stages of disease. In fact, hippocampal atrophy is used as an early biomarker of neuronal injury and to evaluate disease progression. It is not yet well-understood whether changes in hippocampal volume are due to neuronal or glial loss. The aim of the study was to assess hippocampal atrophy and/or gliosis using unbiased stereological quantification and to obtain hippocampal proteomic profiles related to neurodegeneration and gliosis. Hippocampal volume measurement, stereological quantification of NeuN-, Iba-1- and GFAP-positive cells, and sequential window acquisition of all theoretical mass spectrometry (SWATH-MS) analysis were performed in AD and non-AD cases. Reduced hippocampal volume was identified using the Cavalieri probe, particularly in the CA1 region, where it correlated with neuronal loss and astrogliosis. A total of 102 downregulated and 47 upregulated proteins were identified in the SWATH-MS analysis after restrictive filtering based on an FC > 1.5 and *p* value < 0.01. The Hsp90 family of chaperones, particularly BAG3 and HSP90AB1, are closely related to astrocytes, indicating a possible role in degrading Aβ and tau through chaperone-mediated autophagy.

## 1. Introduction

Alzheimer’s disease (AD) is the most prevalent neurodegenerative disorder [1] and is characterized by executive dysfunction and memory impairment [2,3]. Hippocampal volume loss and medial temporal atrophy on magnetic resonance imaging (MRI) have been proposed as early signals of neuronal injury and as diagnostic criteria for AD [4,5,6]. However, MRI studies concerning volume loss in different hippocampal subfields (cornu ammonis fields CA1, CA2, and CA3 and the dentate gyrus, DG) have shown contradictory results. This discrepancy might be explained by the intrinsic difficulty of MRI techniques in delimiting the boundaries of the hippocampal subfields [7,8]. Moreover, histological studies have revealed a global reduction in the hippocampal volume without distinguishing between hippocampal subfields [9,10] or a reduction in hippocampal volume restricted to the CA1 region [11]. On the other hand, postmortem studies using stereological approaches have shown preferential neural loss in the CA1 subfield [11,12,13,14], but neither neural nor glial-specific markers have been used.

Extracellular deposits of amyloid-β peptide (Aβ) and intracellular aggregation of hyperphosphorylated tau are the two major neuropathological features of AD [15,16]. These aggregates appear decades before clinical signs in a specific and predictable manner, allowing the development of a tau-specific six-stage neuropathological diagnostic system (Braak staging): locus coeruleus and hippocampal formation (I–II), limbic structures (III–IV) and isocortical areas (V–VI) [17]. Interestingly, the prion-like hypothesis proposes that both Aβ and tau spread from cell to cell throughout different brain regions [18]. This mechanism includes two steps: seeding (induction of naïve protein misfolding) and spreading (propagation of misfolded proteins transcellularly between neurons and/or glia) [18,19]. This hypothesis is aligned with Braak staging, since different sequentially involved brain regions are hodologically connected [20]. From a connectomic perspective [21], the entorhinal cortex and the hippocampus (HIPP) are crucial hubs for proteinopathy spreading from and to the isocortex [20,22].

The role of neuron–glia interactions in the pathogenesis of AD is now a focus [23], since both microglia [24] and astroglia [25] are involved in proteinopathy propagation [26], as well as synaptic dysfunction [27]. Evidence suggests a protective role for glia in AD, since both glial cell populations participate in the clearance of tau and Aβ (via phagocytosis or production of Aβ-degrading enzymes) [28,29,30]. In this process, the molecular chaperones Hsp70 and Hsp90 [31], and cochaperones, such as BAG3 [32], have emerged as potential regulators of tau and Aβ toxicity. However, recent studies suggest that microglia and astroglia facilitate the spread of pathological proteins and contribute to disease progression [33,34,35]. Unfortunately, data on the involvement of specific neural and glial populations in human hippocampal subfields in AD are very scarce.

Proteomic studies of the human HIPP of the human AD brain have included analyses of human Aβ-enriched extracts [36], studies of microdissected hippocampal fields of diseased [37,38,39] or nondiseased individuals [40], analysis during aging [41], and analysis of the correlation between spatial proteomics of the entire brain and disease severity [42]. However, due to the biological (disease stage, groups of samples, etc.) and methodological variety, the identification of possible AD biomarkers is challenging. Recently, sequential window acquisition of all theoretical mass spectrometry (SWATH-MS) has been developed as a robust proteomic technique that provides not only the broad identification of thousands of proteins, but also a ratio of expression for each one. Different sets of proteins either in cerebrospinal fluid [43] or restricted to the synaptic proteome [44] have been obtained in AD by performing SWATH-MS, establishing a panel of biomarkers with clinical utility and a list of 30 unique synaptic proteins differentially expressed in AD HIPP. Nevertheless, a proteomic analysis of the whole HIPP using SWATH-MS might provide a helpful catalog of protein alterations in the diseased HIPP.

Therefore, the present study aims to characterize the involvement of neurons, microglia, and astroglia in hippocampal subfields using specific markers, constituting the first study where volume and neural and glial populations were estimated in a single report. Stereological data revealed neuronal loss and astrogliosis, specifically in the CA1 region. Moreover, this study comprises the first SWATH-MS analysis of non-AD and AD human HIPP, providing a panel of 1635 identified proteins that is particularly focused on the chaperone family and its association with astroglia.

## 2. Results

### 2.1. Reduction in the Hippocampal Volume

**Figure 1:**
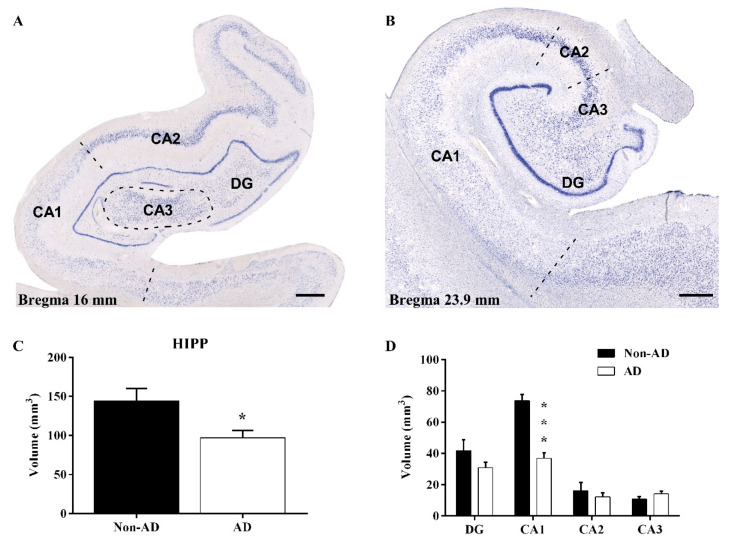
Hippocampal volume reduction is specific to the CA1 subfield. Nissl staining of the AD HIPP at 16 mm (**A**) and 23.9 mm (**B**) from bregma, representing rostral to caudal levels, respectively, and identification of the hippocampal subfields (CA1-3 and the DG). The global HIPP volume (**C**) and volume of the CA1 subfield (**D**) were significantly reduced in AD (the graphs show the volume mean ± SEM, * *p* value < 0.05, *** *p* value < 0.001). Scale bar = 1000 µm.

Nondiseased and diseased human HIPP samples were analyzed from rostral (16 mm from bregma, Figure 1A) to caudal (23.9 mm from bregma, Figure 1B) levels [45]. Nissl-stained sections were used to delimitate the CA1, CA2, CA3, and DG subfields (Figure 1A,B). The Cavalieri probe revealed a significant reduction in global hippocampal volume (unpaired *t* test t16 = 2.596, *p* value = 0.0195) (Figure 1C), as well as a specific reduction in the volume of the CA1 region (unpaired *t* test t16 = 6.803, *p* value < 0.0001) (Figure 1D). The remaining subfields did not show a decrease in volume.

### 2.2. The Extent of Neurodegeneration, Microgliosis and Astrogliosis Differs among Hippocampal Fields

**Figure 2:**
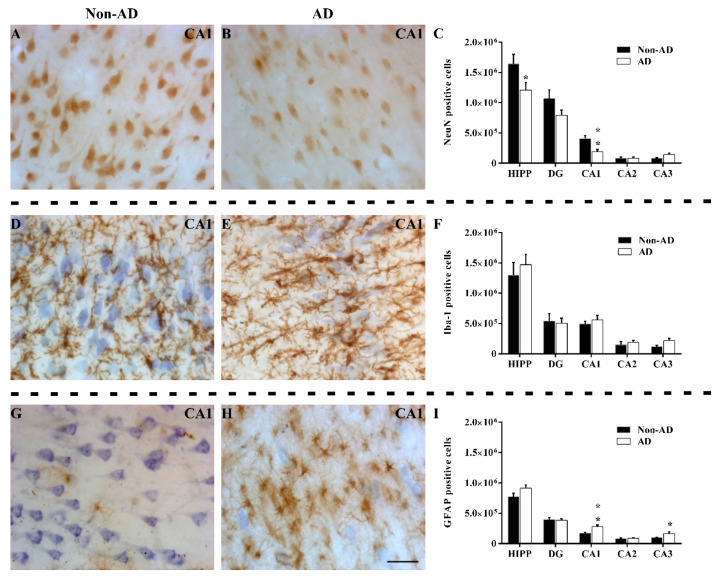
The extent of neurodegeneration, microgliosis, and astrogliosis differs among hippocampal fields. Immunohistochemical staining for NeuN (**A**,**B**), Iba-1 (**D**,**E**), and GFAP (**G**,**H**) in the CA1 subfield in non-AD and AD samples represent neurons, microglia, and astroglia, respectively. The number of NeuN-positive cells (**C**), Iba-1 positive cells (**F**) and GFAP-positive cells (**I**) in the global HIPP and the different subfields are shown (the graphs show the mean ± SEM, * *p* value < 0.05, ** *p* value < 0.01). Please note that neurodegeneration linked to NeuN labeling only occurred in the CA1 region, staining of microglia with Iba-1 was not altered, and GFAP staining of astroglia was increased in both CA1 and CA3. Scale bar = 50 µm.

Quantification of NeuN labeling (Figure 2A,B) showed a global reduction in the number of NeuN-positive cells in the HIPP (unpaired *t* test t16 = 2.167, *p* value = 0.0456), decreasing specifically in the CA1 region (unpaired *t* test t16 = 3.774, *p* value = 0.0017) (Figure 2C). The remaining subfields did not show changes in the number of NeuN-positive cells. Additionally, the intensity of staining in AD samples was noticeably lower than that in non-AD samples (not quantified). The microglial analysis revealed no differences in the number of Iba-1-positive cells (Figure 2D,E) either globally or regionally in the HIPP (Figure 2F). Regarding GFAP quantification (Figure 2G,H), no differences were observed in the whole HIPP. However, a significant increase in the number of GFAP-positive cells in the CA1 (unpaired *t* test t14 = 3.519, *p* value = 0.0034) and CA3 (Mann–Whitney U = 15.00, *p* value = 0.0464) regions (Figure 2I) was reported.

**Figure 3:**
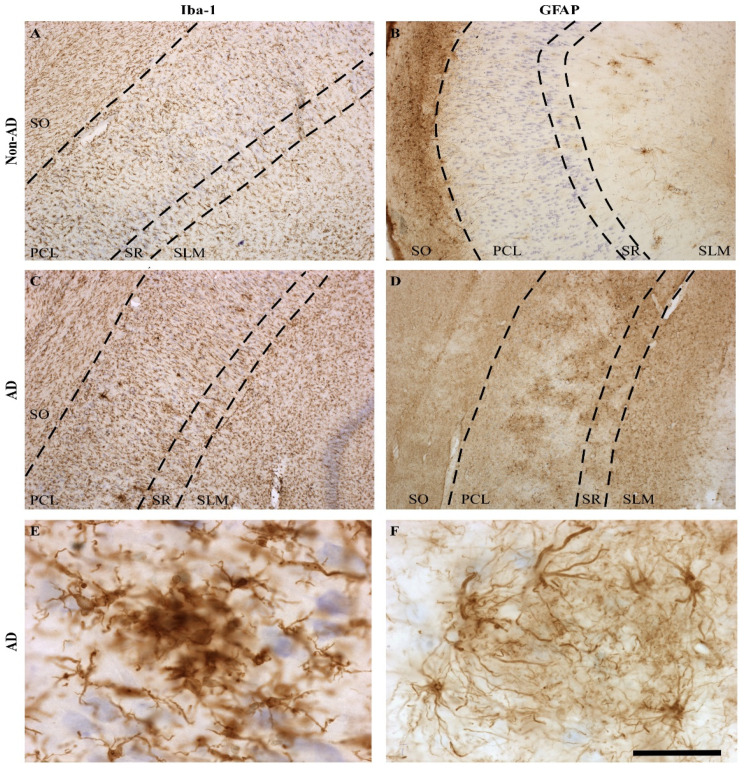
Glial distribution pattern changes in the CA1 region in AD samples. Staining of microglial cells (**A**) revealed homogeneous distribution throughout the different layers in non-AD samples (**A**). Astrocytes (GFAP staining) were mainly found in the SO and SLM layers in non-AD samples (**B**). In AD samples, both microglia and astroglia showed increased labeling and astrocytes reached the PCL and SM layers, forming clusters of cells (**C**,**D**). These clusters were formed by a large number of cells; either microglial cells forming a core (**E**) or astrocytes forming a sphere (**F**). Scale bar = 400 µm in (**A**–**D**); 200 µm in (**E**); and 50 µm in (**F**).

Specifically, in the CA1 region, changes in the morphological features and distribution patterns of microglia and astroglia were observed between the AD and non-AD groups (not quantified). The distribution of microglia throughout layers of the CA1 region was similar between the non-AD (Figure 3A) and AD (Figure 3C) groups. However, microglial cells showed more intense staining and wider ramifications in AD samples. Moreover, clusters of reactive microglia were particularly located in the pyramidal cell layer (PCL) and the *stratum radiatum* (SR) (Figure 3C). These clusters seemed to occupy plaque cores (Figure 3E). On the other hand, both the morphology and distribution pattern of astroglia differed in the layers of the CA1 region between non-AD (Figure 3B) and AD (Figure 3D) samples. In the non-AD group, astroglia were preferentially located in the *stratum oriens* (SO) and occasionally in the *stratum lacunosum moleculare* (SLM) (Figure 3B), whereas in the AD group, astrocytes were mainly located in the PCL, SR, and SLM (Figure 3D). Similar to microglia (Figure 3E), astroglia in the diseased brains formed clusters in the PCL and SR (Figure 3D), but unlike microglia, astroglia clusters appeared around presumptive plaques (Figure 3F).

### 2.3. Expression of Astroglia and Tau in CA1

**Figure 4:**
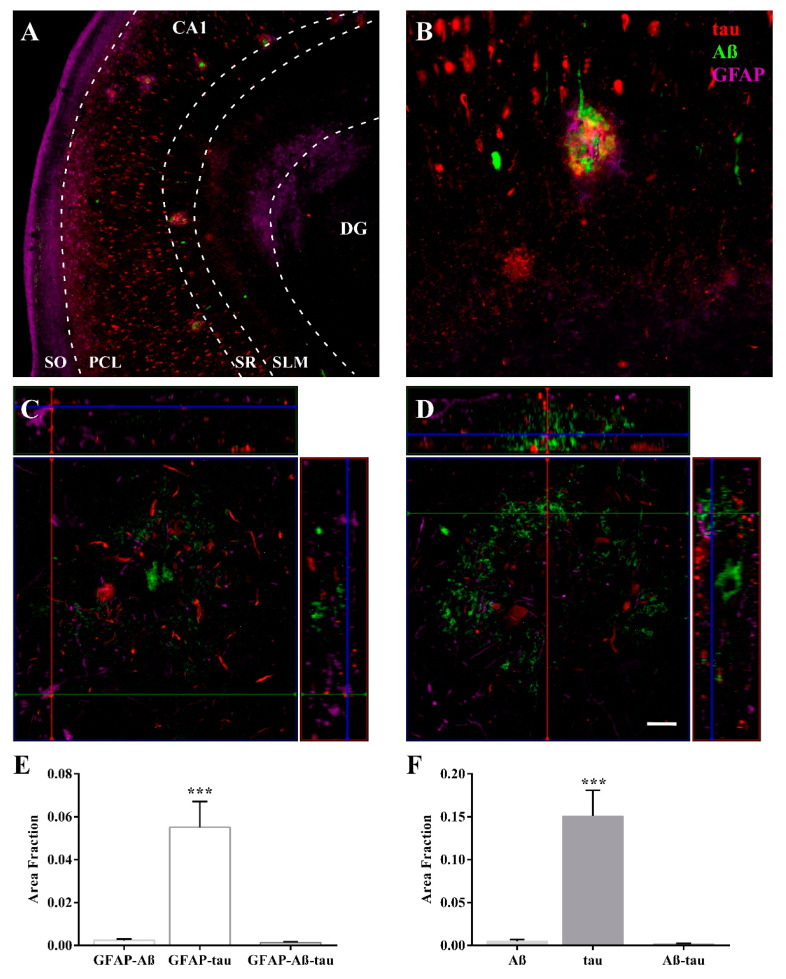
Astrocytes colocalize with tau and form clusters linked with plaques in the CA1 region. Immunofluorescence staining showed preferential distribution of tau in the PCL (**red**) and revealed that Aβ formed plaques in the PCL and SR layers (**green**) and that GFAP was found in all layers of the CA1 region (**violet**) (**A**). Clusters of astrocytes were dispersed around plaques (**B**). Z-stack reconstructions showed astroglial cell bodies (**violet**) around plaques (**C**) and astroglial processes (**violet**) were found in the vicinity of Aβ (**green**) in the plaque core (**D**). The graphs show the area fraction of GFAP-tau colocalization, GFAP-Aβ colocalization, and GFAP-Aβ-tau colocalization (**E**) and the area fraction of tau, Aβ, and both (**F**). (AD group (*n* = 5), the graphs show the mean ± SEM, *** *p* value < 0.001). (**A**,**B**) were obtained with a Zeiss Axio Imager M.2 microscope; (**C**,**D**) were obtained with a Zeiss LSM 800 confocal microscope. Scale bar = 250 µm in (**A**), 50 µm in (**B**), and 10 µm in (**C**,**D**).

After assessing the astrocyte response in the CA1 region, we decided to analyze the spatial relationship of astrocytes with pathological markers. GFAP staining generally overlapped with pathological tau protein expression. An analysis of the distribution and colocalization of either GFAP and tau or GFAP and Aβ was performed using the area fraction fractionator (AFF) method. As described above, astrocytes were predominantly located in the SLM (Figure 4A) encircling plaques (Figure 4B). Confocal microscopy showed the presence of Aβ in the core, tau in the periphery, and astrocytes surrounding plaques. Astroglial bodies encircled plaques interacting with tau (Figure 4C), whereas astroglial processes reached the plaque core in the vicinity of Aβ (Figure 4D, the Z-stack reconstruction is shown in Supplementary Materials Video S1). Quantification using the AFF method revealed a greater spatial colocalization of astrocytes and tau than Aβ or even tau plus Aβ (Figure 4E) (one-way ANOVA F (2, 12) = 19.62, *p* value = 0.0002). Furthermore, quantification of pathological protein expression using the AFF method revealed that the tau-positive area was larger than the Aβ-positive area (Figure 4F) (one-way ANOVA F (2, 12) = 23.92, *p* value < 0.0001).

### 2.4. Proteomic Analysis Highlights the Involvement of HSP90 Family cChaperones

**Figure 5:**
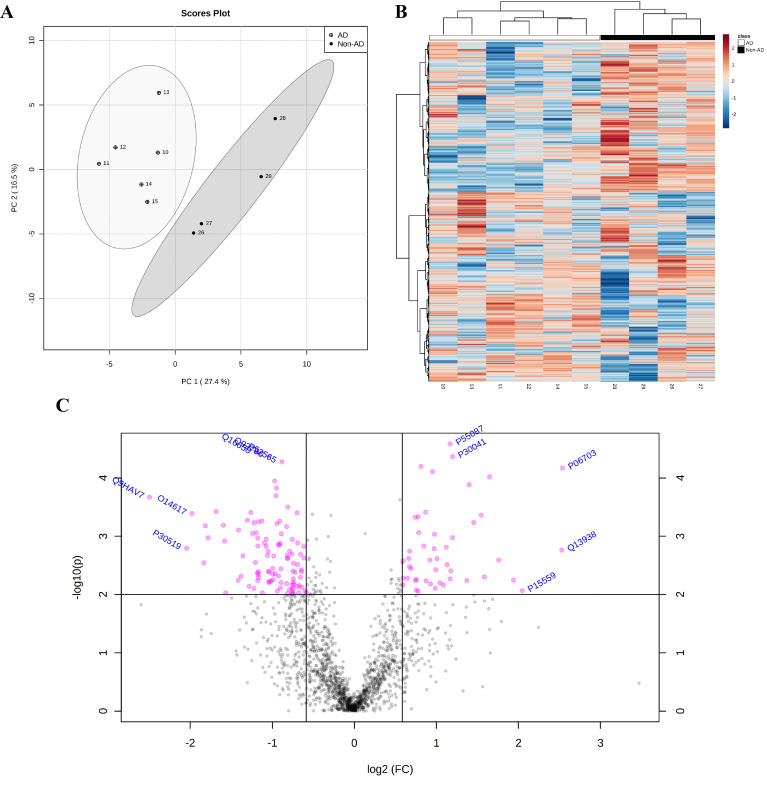
Prominent downregulated proteins in AD samples. Principal component analysis (PCA) revealed two-well defined groups of AD and non-AD samples (**A**). The cluster analysis data visualized by heatmap reveal the differential expression of 1635 proteins between groups (**B**). The volcano plot shows 47 up- and 102 downregulated proteins among a total of 1635 analyzed proteins (**C**) (FC > 1.5, *p* value < 0.01). Statistical analysis was performed with MetaboAnalyst 5.0.

The SWATH-MS analysis identified 1635 proteins. Principal component analysis (PCA) and heatmap analysis revealed two well-defined groups after logarithmic transformation (Figure 5A,B). A total of 102 downregulated and 47 upregulated proteins were recognized after restrictive filtering using an FC > 1.5 and *p* value < 0.01, as illustrated in the volcano plot (Figure 5C) (for a detailed list of all identified up/downregulated proteins, see Supplementary Materials Table S2). Gene Ontology (GO), protein–protein interactions (PPIs) and pathway analyses of all listed proteins (149 up/downregulated proteins) were performed (for Gene Ontology analysis see Supplementary Materials Table S3; for pathway analysis, see Supplementary Materials Table S4).

**Figure 6:**
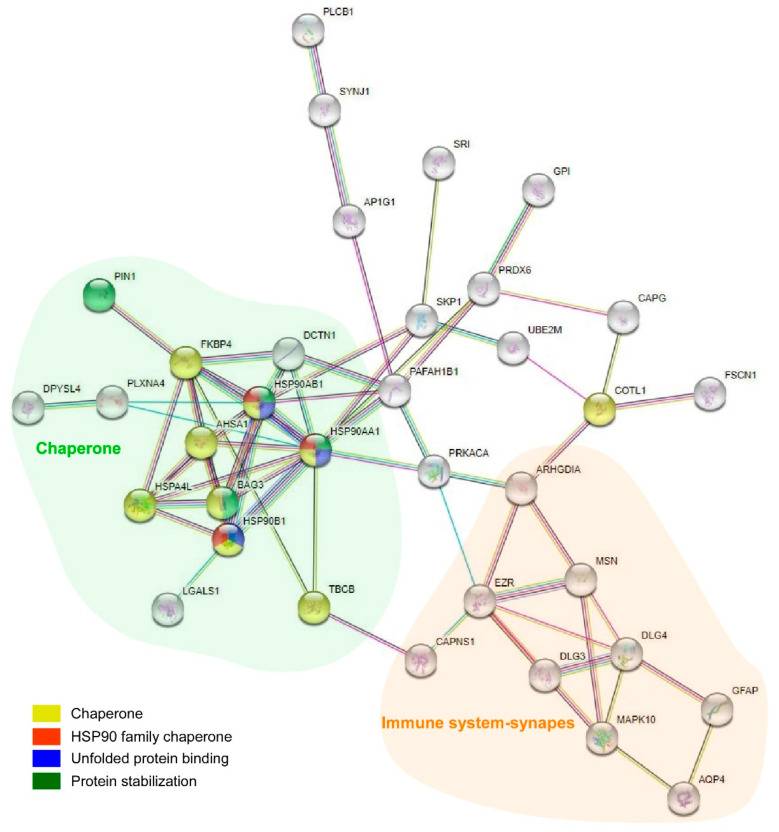
Protein–protein interaction (PPI) analysis revealed a complex interaction between proteins, with clusters related to astrocytes, synapses, and chaperones. Immune system-/synapse-related proteins are within the orange cloud, and chaperones are within the green cloud. Proteins that belong to the Hsp90 family of chaperones are highlighted in red, genes enriched in the molecular function unfolded protein binding are highlighted in blue, and genes enriched in the biological process protein stabilization are highlighted in green. Note that HSP90AB1 and HSP90AA1 are involved in all identified functions in the analysis. In addition, BAG3 is related to protein stabilization.

The GO analysis identified the Hsp90 family of chaperones as a protein class (fold enrichment: 51.83), highlighting unfolded protein binding as the main molecular function (fold enrichment: 10.05) and the biological process of protein stabilization (fold enrichment: 29.10) (Table 1). In parallel, the PPIs data were evaluated to identify clusters of interest and indicated a connection between astrocytes, synapses, and chaperones (36 proteins involved) (Figure 6). Proteins involved in the connection between the chaperones, immune system, and synapse were evaluated. Nine proteins were identified as chaperones (HSPA4L, FKBP4, HSP90AA1, HSP90AB1, BAG3, HSP90B1, AHSA1, TBCB, and COTL1) (Figure 6, yellow), three of which were members of the HSP90 family of chaperones (HSP90B1, HSP90AA1, and HSP90AB1) (Figure 6, red); three proteins were associated with unfolded protein-binding (HSP90AA1, HSP90AB1, and HSP90B1) (Figure 6, blue) and four proteins were associated with protein stabilization (PIN1, BAG3, HSP90AA1, and HSP90AB1) (Figure 6, green). After considering these data, HSP90AA1 and HSP90AB1 were identified as proteins of interest.

Proteins recognized as chaperones were subjected to a Reactome analysis to identify the involved pathways (Table 2). A prominent pathway was chaperone-mediated autophagy (R-HSA-9613829), which exposed the interactions of HSP90AA1 and HSP90AB1 with GFAP. In addition, the innate immune system (R-HSA-168249; HSP90AA1, COTL1, HPS90B1, and HSP90AB1), signaling by interleukins (R-HSA-449147; HSP90AA1 and HSP90B1), and cellular responses to external stimuli (R-HSA-8953897; HPS90AA1, HSPA4L, FKBP4, BAG3, and HSP90AB1) were identified in the analysis.

Based on proteomic data and their possible relationship with astrocytes in the disease, BAG3, HSP90AA1, HSP90AB1, HSPA4L, and FKBP4 were selected for further immunofluorescence and Western blot (WB) analyses to assess the relevance of heat shock proteins (HSPs) in AD [46]. Nonconclusive results were obtained for HSPA4L and FKBP4 expression.

### 2.5. Differential Colocalization of Chaperones with Astrocytes

**Figure 7:**
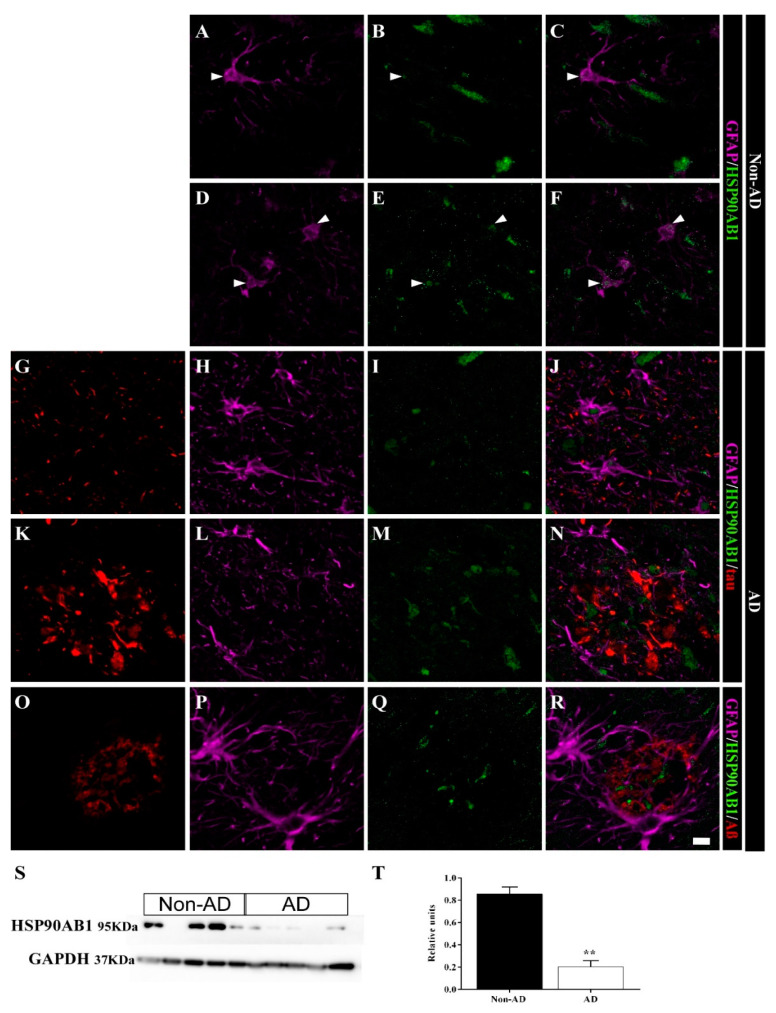
HSP90AB1 is colocalized with astrocytes in the human HIPP. Confocal analyses of GFAP (**violet**), HSP90AB1 (**green**) and tau or Aβ (**red**) expression in the CA1 region in non-AD (**A**–**F**) and AD (**G**–**R**) samples. In non-AD cases, HSP90AB1 was observed in astrocytes (**arrowhead**) (**A**–**F**). In AD samples, HSP90AB1 was found in plaque core, but was not expressed in astrocytes in the vicinity (**N**,**R**). However, isolated astrocytes showed a similar expression pattern between AD samples and non-AD samples (**J**). HSP90AB1 expression throughout the whole human HIPP was evaluated by WB (**S**,**T**) (the graph shows the mean ± SEM, ** *p* value < 0.01). Scale bar = 10 µm.

The expression of the chaperone HSP90AB1 was downregulated in the HIPP of AD samples according to the SWATH-MS analysis (FC = 0.50, *p* value = 0.00447). Immunofluorescence staining revealed that HSP90AB1 was expressed in astrocytes and presumably in neurons in non-AD samples (Figure 7A–F). Astrocytes in AD samples (Figure 7G–J) also expressed this chaperone protein, similar to non-AD samples. However, astrocyte cell bodies in the vicinity of plaques did not express this protein (Figure 7K–R). In contrast, HSP90AB1 was detected in the cores of plaques close to astrocyte processes (Figure 7N,R). HSP90AB1 downregulation was confirmed by the WB analysis (Figure 7S,T) (unpaired *t* test t4 = 7.834, *p* value = 0.0014).

**Figure 8:**
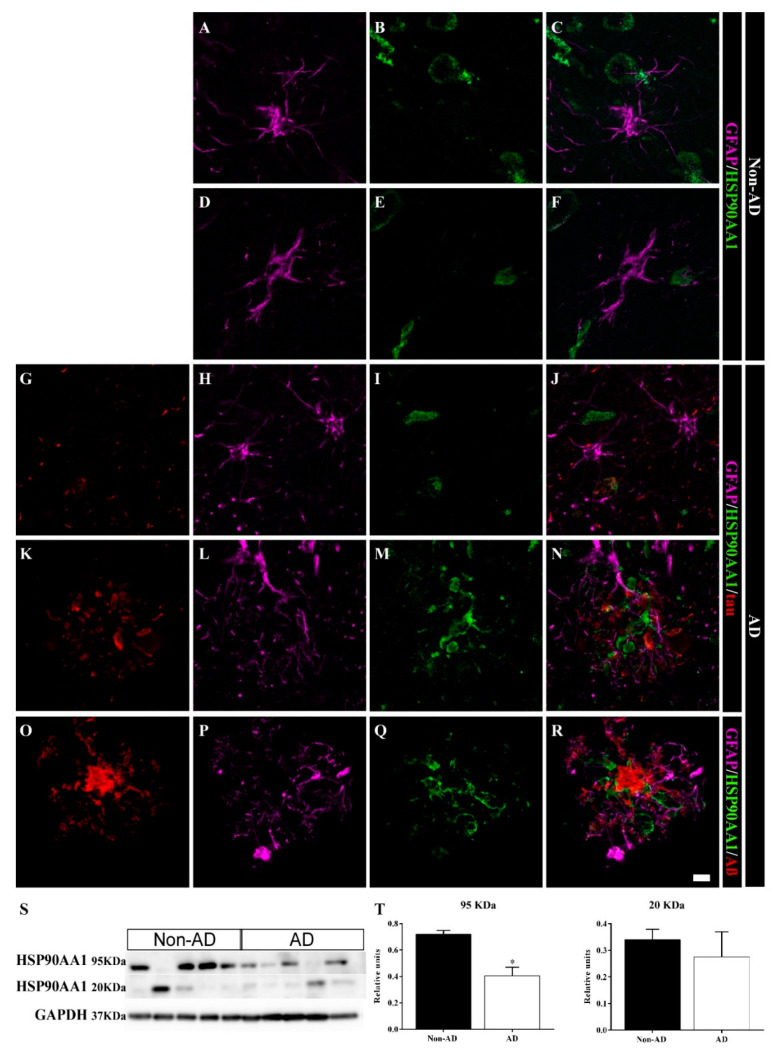
HSP90AA1 accumulates within plaques in the human HIPP. Confocal images of GFAP (**violet**), HSP90AA1 (**green**) and tau or Aβ (**red**) expression in the CA1 region in non-AD (**A**–**F**) and AD (**G**–**R**) samples. In AD cases, HSP90AA1 aggregated in plaques (**N**,**R**), and was in close contact with the Aβ plaque core (**R**). Astrocytes did not express HSP90AA1 in non-AD (**A**–**F**) or AD (**K**–**O**) samples. (**S**,**T**) HSP90AA1 expression throughout the whole human HIPP was evaluated by WB (the graph shows the mean ± SEM, * *p* value < 0.05). Scale bar = 10 µm.

According to the SWATH-MS analysis, the expression of the chaperone HSP90AA1 was downregulated in the HIPP of AD samples (FC = 0.63, *p* value = 0.00701). In contrast to HSP90AB1, astrocytes did not express HSP90AA1 in non-AD samples (Figure 8A–F). In AD samples, neither isolated astrocytes (Figure 8G–J) nor those located in the vicinity of the plaques (Figure 8K–R) expressed this chaperone. On the other hand, HSP90AA1 was identified in the cores of the plaques near astrocyte processes (Figure 8N,R) and was located closer to Aβ (Figure 8R) than to tau (Figure 8N). WB analysis showed two isoforms, a 95 kDa isoform and a 20 kDa isoform, of HSP90AA1 (Figure 8S). Only the 95 kDa band showed decreased expression in AD samples (unpaired *t* test t4 = 4.354, *p* value = 0.0121) (Figure 8T). Curiously, two samples that did not express the 95 kDa isoform showed marked expression of the 20 kDa isoform. The first sample was a non-AD sample (case 26, Table 3), and the other sample was an AD sample (case 12, Table 3), which corresponded to Braak II and Braak VI + Lewy body dementia (LBD) cases, respectively.

**Figure 9:**
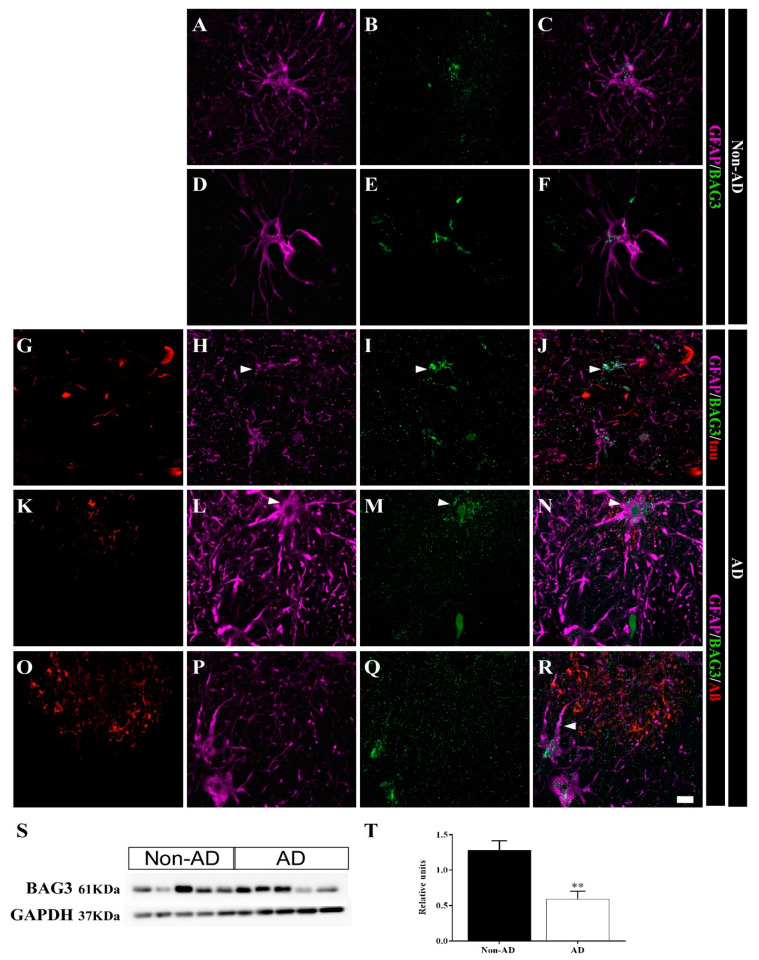
BAG3 is expressed in astrocytes in the human HIPP. Confocal analyses of GFAP (**violet**), BAG3 (**green**) and tau or Aβ (**red**) expression in the CA1 region in non-AD (**A**–**F**) and AD (**G**–**R**) samples. BAG3 was found in astrocytes in both non-AD samples (**A**–**F**) and overexpressed in AD samples (**G**–**R**). Some astrocytes showed mainly BAG3 staining instead of GFAP staining (**H**–**J**, arrowhead). BAG3 released within and around plaques was frequently observed (**K**–**R**). (**S**,**T**) BAG3 expression throughout the whole human HIPP was evaluated by WB (the graph shows the mean ± SEM, ** *p* value < 0.01). Scale bar = 10 µm.

The expression of the cochaperone BAG3 was upregulated in the HIPP of AD samples according to the SWATH-MS analysis (FC = 2.64, *p* value = 0.00013). Confocal analysis revealed BAG3 expression in astrocytes in both non-AD (Figure 9A–F) and AD samples (Figure 9G–R). Moreover, astrocytes in AD samples were identified solely by BAG3 staining, and GFAP staining was reduced in these cells (Figure 9G–J, arrowhead). Astrocytes overexpressing BAG3 were commonly detected surrounding plaques (Figure 9K–N, arrowhead). Furthermore, BAG3 expression was frequently observed in association with processes (Figure 9R, arrowhead) and dispersed as aggregates within the cores of Aβ plaques (Figure 9R). In contrast to the SWATH-MS results, WB analysis showed an unexpected decrease in BAG3 expression in AD samples (unpaired *t* test t6 = 3.929, *p* value = 0.0077) (Figure 9S,T).

## 3. Discussion

Stereological results showed reduced hippocampal volume in AD, which was limited to the CA1 region. Furthermore, neuronal loss was specifically restricted to the CA1 region and was accompanied by increased astrogliosis in this subfield. In the AD HIPP, particularly in the CA1 region, astrocytes were dispersed in clusters throughout the PCL. Cell bodies were located in the periphery surrounding the plaques, whereas their ramifications reached the core. According to the proteomics results, chaperones were the main protein class identified in the analysis. In particular, they were involved in pathways such as chaperone-mediated autophagy, and a possible interaction between these proteins and astrocytes was indicated. Confocal analysis revealed downregulation of HSP90AB1 expression and upregulation of BAG3 expression in astrocytes from non-AD samples. In addition, these chaperones or cochaperones were coexpressed with both pathological proteins, mainly tau, and astrocytes in AD samples, suggesting their participation in the disease.

### 3.1. Volume Reduction, Neurodegeneration and Astrogliosis Occur Specifically in the CA1 Region

Hippocampal atrophy has been highlighted as a biomarker of neuronal injury [4] and a predictor of AD progression [47], and is increasingly used as an indicator for clinical diagnosis [6]. Consistent with most studies [12,13,48], our results suggested marked neurodegeneration in the CA1 region associated with astrogliosis and a reduced volume. However, studies performing a stereological assessment of the involvement of glia have been limited to some subfields [49] or have not distinguished between hippocampal subfields [50]. The increase in astrogliosis was tightly related to neurodegeneration and pathological protein expression in the CA1 region, as well as the distribution pattern of glia. Astrocytes and microglia showed a similar distribution since they were grouped in clusters in the PCL and SR. As described in other studies [51,52], microglia were found within plaques, whereas astrocytes surrounded them. In non-AD samples, astroglia were predominantly located in the SO and SLM, whereas in AD samples, increased astroglial immunoreactivity was observed mainly in the PCL and SR. Astrocyte activation might occur as a response to the expression of pathological proteins, since Aβ deposits and hyperphosphorylated tau are located mainly in the PCL and SR in the human AD HIPP. In addition, astrocytes showed marked arborization, and cell bodies were intensely stained in AD samples. Therefore, both the number of astrocytes and GFAP expression might be increased. Further studies are needed to investigate whether these changes constitute a neuroprotective strategy or exacerbate neurodegeneration [25].

The association of glial cells with pathological proteins has been evaluated separately for Aβ and tau [51], and the association of pathological proteins centered in plaques with astrocytes has also been evaluated [53]. Contradictory roles for astrocytes have been described, as the formation of glia-associated plaques has been suggested to be an early event contributing to AD [53] and to occur in the late stages of the disease [51]. As described above, plaques composed of an Aβ core and tau were located mainly in the PCL and SR. In addition, tau rather than Aβ occupied most of the CA1 area, and colocalization between astrocytes and tau was observed. This finding might explain why astrocytes distributed in the PCL and SR colocalized with tangles, whereas only astrocyte processes reached the Aβ core. Additionally, astrocytes are known to internalize tau released into the extracellular space by neurons [54]. Regarding internalization, astrocytes degrade assimilated tau, and release tau from the cell to lead to its potential propagation and intracellular accumulation, generating an inflammatory cytokine response. However, the mechanisms of tau transport into and out of astrocytes in AD remain unknown [55].

Therefore, the CA1 region is proposed as the main hub underlying hippocampal involvement in AD, which might be related to hippocampal formation connections. The entorhinal cortex is one of the first affected regions in subjects with AD, and it is tightly connected to the HIPP through the perforant pathway (layer II-DG/CA3) and projections from layer III to CA1 via the subiculum. Moreover, the DG connects to the CA3 through mossy fibers, and finally, Schaffer collaterals connect the CA3 region with the CA1 region [56,57]. Considering these connections, the CA1 region is an endpoint where two pathways of connections between the entorhinal cortex and the HIPP converge, suggesting that the CA1 region is doubly vulnerable to pathological changes [20]. Interestingly, the capability of the pathological proteins Aβ and tau to spread in a prion-like manner may particularly contribute to the particular vulnerability of the CA1 region.

### 3.2. HSP90AB1 and BAG3 Expression in Astrocytes Indicates Possible Roles in Aβ and Tau Homeostasis

Protein aggregation is the main hallmark of many neurodegenerative disorders [58]. Chaperones redirect aggregated proteins to the monomeric form, remodel oligomers into a less toxic form, inhibit some steps in the protein aggregation process, and target aggregates for degradation through autophagic and proteosomal processes [59]. Chaperones and cochaperones have been discussed as potential therapeutic targets for AD, Parkinson’s disease, and Huntington’s disease, among other diseases [60]. Moreover, FKBP51, a cochaperone of HSP90, has been proposed as a possible biomarker and therapeutic target for mental disorders [61].

Among 149 up-/downregulated proteins, the Hsp90 family of chaperones was highlighted as playing an important role in HIPP-related disease. HSP90 is the chaperone most frequently detected in neurons and is the major regulator of protein-folding in cells [60]. In mammals, two major cytoplasmic isoforms of HSP90 are expressed: HSP90β (HSP90AB1), which is constitutively expressed, and HSP90α (HSP90AA1), which responds to stress conditions and is involved in protein transport and folding [62]. Accumulating evidence regarding the roles of HSP90 and its cochaperones in the folding and degradation of pathological proteins in neurogenerative diseases has been obtained in the last decade [60,63]. According to the SWATH-MS analysis, HSP90AB1 and HSP90AA1 expression is downregulated in the AD HIPP. In addition, the WB analysis of HSP90AA1 expression revealed two bands that might correspond to the 95 kDa and 20 kDa isoforms of HSP90AA1 (UniProt-G3V2J8). The WB analysis of HSP90AB1 and HSP90AA1 expression confirmed that the expression of both proteins was downregulated in the AD HIPP, and a similar result was also obtained from the confocal analysis. In AD cases, HSP90 expression was reduced in CA1 neurons (not quantified), consistent with the neurodegeneration observed in this region. Although both isoforms accumulated in plaques, their distribution differed slightly. HSP90AA1 was distributed preferentially in the cores of plaques close to Aβ, whereas HSP90AB1 was expressed not only in neurons, but also in astrocytes encircling plaques. In AD, the HSP90 and HSP70/HSP40 complex inhibits Aβ oligomerization and slows the rate of aggregation [64]. In addition, inhibition of HSP90 in both cellular and mouse models of tauopathies leads to a reduction in the pathogenic activity and elimination of aggregated tau [65]. HSP90AB1 is released into the extracellular space by astrocytes, where it interacts with tau and promotes the degradation process, exerting a neuroprotective effect. In contrast, the rerelease of tau associated with chaperone activity might exert a neurotoxic effect [55]. Therefore, HSP90 has been suggested as a possible therapeutic target, since its inhibition has been shown to reduce tau levels and decrease the toxicity induced by Aβ [66].

On the other hand, BAG3, a cochaperone that binds to heat-shock cognate (HSC) 70/HSP70 and HSPB8, specifically regulates protein degradation by autophagy [67]. In AD, BAG3 plays a fundamental role in regulating the levels of tau in neurons by activating autophagy [68]. Furthermore, upregulation of BAG3 expression has been proposed as a therapeutic strategy for AD [69]. Increased expression of BAG3 was observed in the AD HIPP using the SWATH-MS analysis, which differed from the WB results showing a reduction in BAG3 expression. This discrepancy may be explained by the different approaches employed in each analysis: the SWATH-MS analysis identifies protein-associated peptides, and the complete protein is detected using WB analysis. BAG3 integrity might be compromised by the pathological conditions in AD, preventing it from being detected in the WB analysis. Nevertheless, BAG3 expression in astrocytes was assessed using confocal microscopy, and BAG3 was particularly visible in AD samples, where astrocytes were identified not only by GFAP but also by BAG3 expression. In addition, BAG3 was detected in the extracellular space, where it formed aggregates but was not clearly colocalized with pathological proteins. Upregulation of BAG3 expression in astrocytes in the entorhinal cortex in postmortem tissue from subjects with AD, whereas no differences were detected in neurons, suggesting the ability of astrocytes to clear aggregated tau and/or Aβ released from neurons and cellular debris [70].

Chaperone-mediated autophagy dysfunction has been proposed to be involved in the pathogenesis of neurodegenerative diseases, and its role as a potential therapeutic target is being discussed [71]. Astrocytes may participate in AD pathology by expressing and/or releasing chaperones and cochaperones (HSP90AB1 and BAG3) to mediate the autophagic clearance of tau and Aβ aggregates. However, this study provides a static image of the situation in the last stages of the disease; therefore, further research on early stages of the disease and research using in vitro and in vivo approaches is needed.

## 4. Materials and Methods

### 4.1. Human Samples

Samples and data were provided by IDIBAPS, BIOBANK-MUR, BTCIEN, and BPA, integrated in the Spanish National Biobanks Network, and processed according to standard operating procedures after obtaining approval from the appropriate the Ethics and Scientific Committees. These protocols included obtaining written consent from the donors. Experimental procedures were approved by the Ethics Committee of Clinical Research of Ciudad Real University Hospital (SAF2016-75768-R and PID2019-108659RB-I00).

Thirty-two cases were selected for the study (Table 3): 16 were diagnosed with Alzheimer’s disease (AD), and 16 were classified as non-AD. No differences between the AD and non-AD groups in age, postmortem delay, or brain weight were observed, except for a difference in brain weight between samples analyzed using SWATH-MS (unpaired *t* test t8 = 3.168, *p* value = 0.0132; for details, see Supplementary Materials Figure S1). Formalin-fixed samples were employed for immunohistochemistry and stereological quantifications (*n* = 18, AD *n* = 9, non-AD *n* = 9). Fresh-frozen samples were used for the WB analysis (*n* = 10, AD *n* = 5, non-AD *n* = 5) and SWATH-MS analysis (*n* = 10, AD *n* = 6, non-AD *n* = 4).

Formalin-fixed samples from different tissue banks were postfixed with fresh phosphate-buffered 4% paraformaldehyde for 45 days. For cryoprotection, blocks were immersed for 48 h in a phosphate-buffered (PB) solution containing 2% dimethyl sulfoxide (DMSO) and 10% glycerol and for 48 h in a PB solution containing 2% DMSO and 20% glycerol. A freezing sliding microtome was used to obtain 50-μm-thick coronal sections. Thirteen series were obtained from each block, and the distance between sections was 650 μm. The first series was used for Nissl staining. The remaining series were stored in 24-well plates at −20 °C in 30% ethylene glycol and 20% glycerol in 0.1 M PB (pH 7.4).

### 4.2. Immunoperoxidase Immunohistochemistry

Tissue epitopes were unmasked by boiling the tissue in citrate buffer under pressure for 2 min. The sections were immersed in formic acid for 3 min and rinsed with phosphate-buffered saline (PBS). Endogenous peroxidase activity was inhibited by incubating the samples in 1% H_2_O_2_ in PBS for 20 min. Sections were preincubated for 1 h (NeuN and Iba-1) or 2 h (GFAP) with blocking buffer and incubated overnight at 4 °C with primary antibodies (NeuN, Iba-1 and GFAP) (for details, see Supplementary Materials Table S1). The sections were then incubated with a biotinylated anti-rabbit secondary antibody (1:200; Vector Laboratories) for 2 h at room temperature and in avidin–biotin complex (ABC Standard; Vector Laboratories) and reacted with 0.025% 3.3′-diaminobenzidine and 0.1% H_2_O_2_. The sections were mounted, counterstained with Nissl, dried, dehydrated, and coverslipped with DPX (Sigma–Aldrich, Saint Louis, MI, USA).

### 4.3. Immunofluorescence Immunohistochemistry

Similar to immunohistochemistry, tissue epitopes were unmasked and subsequently exposed to UV light for 24 h to reduce autofluorescence. Sections were incubated with blocking buffer for 2 h and with primary antibodies (tau, Aβ and GFAP) for 72 h at 4 °C twice. In addition, for staining with HSP90AA1, HSP90AB1 and BAG3 antibodies, the sections were incubated with blocking buffer for 1 h and with the antibodies for 48 h or 24 h at 4 °C (for details, see Supplementary Materials Table S1).

Subsequently, the sections were incubated with Alexa Fluor 594-conjugated anti-rabbit, Alexa Fluor 488-conjugated anti-mouse or Alexa Fluor 647-conjugated anti-goat antibodies (1:200; Thermo Fisher, Waltham, Massachusetts, USA) for 2 h and then with 0.05% DAPI for 10 min at room temperature. Sections were mounted and coverslipped with PVA-DABCO.

### 4.4. Stereological Quantifications

The human hippocampal volume and neuronal, microglial, and astroglial populations were quantified using a Zeiss Axio Imager M.2 microscope coupled to stereological software (StereoInvestigator, MBF Bioscience^®^, Williston, VT, USA). Four sections from each tissue spanning a length of 2.6 mm of the HIPP along the rostrocaudal axis from 16 to 23.9 mm from the bregma were selected for quantification [45]. The same number of sections (*n* = 4) from regular intervals (650 µm) at each bregma level per case and group were selected for analysis. The hippocampal subfields were delimited with a 1× objective (Zeiss Plan-Neofluar 1×/0.025, Ref. 420300-9900), and quantification was performed with a 63× objective (Zeiss Plan-Apochromat 63×/1.4 oil DIC, Ref. 420782–9900) (Figure 1) [72].

Volumes were estimated using the Cavalieri estimator probe. The numbers of NeuN-, Iba-1- and GFAP-expressing cells were quantified using the optical fractionator method, and the tau-, Aβ- and GFAP-positive areas were assessed with the AFF method. The dissector height (Z) was 9 µm, and the guard zones were 2 µm. The area fraction showing GFAP-tau colocalization, GFAP-Aβ colocalization, GFAP-tau-Aβ colocalization, tau expression, Aβ expression, and tau-Aβ colocalization in the AD samples was determined using hippocampal mosaics obtained with a 20× objective (Zeiss Plan-APOCHROMAT 20×/0.8), Ref. 420650-9901). All acquired data presented a coefficient of error (Gundersen), m = 1 < 0.1 (for details, see the Supplementary Materials, File S1).

### 4.5. Immunoblotting

Frozen samples were homogenized in RIPA buffer (50 mM Tris-HCl, pH 7.4, 150 mM NaCl, 0.1% Triton X-100, 0.1% SDS, and 0.5% Na-deoxycholate) using micropestles and then incubated for 2 h at 4 °C. Protein extraction was performed by centrifugation at 12,000× *g* for 5 min at 4 °C, and the supernatant was collected. The protein concentration was measured using the bicinchoninic acid method (BCA).

Thirty micrograms of each protein sample were loaded on 10% SDS-Tris-Trizma-PAGE and transferred to nitrocellulose (HSP90AA1) or PVDF (HSP90AB1, BAG3) membranes (Bio–Rad, Hercules, California, USA). The membranes were blocked for 1 h with 5% low-fat milk in 0.1% Tween-20, 0.06 M NaCl, and 0.2 M Tris-hydroxymethyl-aminomethane (pH 8.8) (TTBS) and incubated overnight at 4 °C with the appropriate antibody (for details, see Supplementary Materials Table S1). The membranes were then incubated with horseradish peroxidase-conjugated secondary antibody (Eurogentec, Cultek, Madrid, Spain) diluted 1:5000. The blots were developed using the ECL-plus detection method (HSP90AB1, BAG3, GAPDH) or SuperSignal West Femto Maximum Sensitivity Substrate (HSP90AA1) (Thermo Fisher Scientific, Waltham, MA, USA). GAPDH was used as housekeeping protein. Images were obtained with the Syngene G:Box system (GeneSys Software, Daly City, CA, USA) and then analyzed using ImageJ software (Fiji, free software). The WB results are shown as the means ± SEM of three independent experiments.

### 4.6. Proteomic Analysis

#### 4.6.1. Sample Preparation

Protein extracts were obtained from the human tissue samples, and protein was precipitated with TCA/acetone to remove contaminants and resuspended in 0.2% RapiGest SF (Waters, Milford, Massachusetts, Estados Unidos). The total protein concentration was measured with a Qubit fluorimetric protein assay (Thermo Fisher Scientific, Waltham, MA, USA). Forty micrograms of protein from each sample were digested with trypsin, and massive protein relative quantitation was conducted using the SWATH-MS approach, as previously described [72].

#### 4.6.2. Protein-Peptide-MS/MS Library Building

Briefly, a tandem mass spectrometry (MS/MS) peptide library was built from the peptides and proteins identified using data-dependent acquisition (DDA) shotgun nano LC–MS/MS analyses of the samples. The MS/MS spectra of the identified peptides were then used to generate the spectral library for SWATH peak extraction using the add-in for PeakView Software (version 2.1, Sciex, Connecticut Path Framingham, MA, USA) MS/MSALL with the SWATH Acquisition MicroApp (version 2.0, Sciex, Connecticut Path Framingham, MA, USA). Peptides with a confidence score greater than 99% determined with the Protein Pilot database search were included in the spectral library. The detailed LC and MS parameters that were used in the present study are provided in the Supplementary Materials, File S2.

#### 4.6.3. SWATH Data Acquisition and Analysis

Each sample was analyzed with a variable SWATH LC–MS method using the same LC–MS system and gradient, as used for the previous DDA runs but using SWATH data-independent acquisition (DIA). The MS parameters used for SWATH are described in detail in the Supplementary Materials, File S2. Quantitative information for the proteins contained in the library was obtained from the SWATH runs by extracting the corresponding fragment ion chromatograms using MS/MSALL with SWATH Acquisition MicroApp. Peptide retention times were calibrated in all SWATH runs using endogenous peptides from an abundant protein (18 peptides from P14618, pyruvate kinase). Up to 10 peptides per protein and seven fragments per peptide were selected based on signal intensity; any shared and modified peptides were excluded from processing. Only those peptides showing confidence scores greater than 95% and a false discovery rate (FDR) less than 1% were used for protein quantitation, which was calculated by adding the chromatogram areas of the corresponding peptides, to ensure confidence in the proteins being quantified.

### 4.7. Pathway Analysis

For the proteomic analysis, logarithmic transformation and t tests were applied. PCA, heatmap, and volcano plots were generated with MetaboAnalyst 5.0. The Gene Ontology Panther tool was used to identify biological processes, molecular functions, cellular components, and protein classes. Pathway analysis and PPIs were obtained with Reactome and STRING, respectively. A fold change (FC) > 1.5 for upregulated expression and an FC < 0.67 for downregulated expression and a *p* value < 0.01 were used for Gene Ontology, pathway and PPIs analyses.

### 4.8. Confocal Analysis

Triple immunofluorescence staining of pathological proteins, GFAP and proteins identified by the SWATH-MS analysis was analyzed with a Zeiss LSM 800 confocal microscope coupled to Zen 2.3 software, Oberkochen, Germany. Spatial colocalization was analyzed in high magnification images obtained with a 63× objective (Zeiss Plan-Apochromat 63×/1.4 Oil DIC M27-oil), Ref. 420782-9900-799). Z-stacks were acquired to evaluate the colocalization of identified proteins within plaques and astrocytes.

### 4.9. Statistical Analysis

Statistical analyses were performed with GraphPad Prism 6 software. For stereological quantifications, the normality of the data was assessed using the Shapiro–Wilk test. The data are presented as the means ± SEM. For normally distributed data, mean values were compared using *t* tests, and the Mann–Whitney U test was used for nonnormally distributed data. F tests were performed to compare variances, and *t* tests with Welch’s correction were performed when differences between variances were observed. The ROUT method was employed to identify outliers. A significance level α = 0.05 was used.

## 5. Conclusions

To the best of our knowledge, this study is the first to use stereology to estimate the volume of hippocampal subfields and analyze neurons and glia using cell type-specific markers in these subfields in postmortem tissue from subjects with AD. Possible neurodegeneration and increased astrogliosis in the CA1 region indicate that it is the most vulnerable region to pathological changes. The proteomic results highlighted the possible role of astrocytes in chaperone-mediated autophagy of pathological Aβ and tau. Additionally, we have shown for the first time that HSP90AB1 is expressed in astroglia in the human HIPP. Both HSP90AB1 and BAG3 are present in astrocytes, indicating their potential involvement in tau and Aβ homeostasis. Therefore, due to their participation in the regulation of pathological protein levels, HSP90 and BAG3 should be considered interesting therapeutic targets for AD. Further studies focused on the probable mechanism of chaperone-mediated autophagy in astrocytes would help to develop valuable treatment approaches to slow pathological progression.

## Figures and Tables

**Table 1:** Gene Ontology analysis †.

	Fold Enrichment	Raw *p* Value	FDR
**PROTEIN CLASS**			
**Hsp90 family chaperone (PC00028)**	**51.83**	**5.74 × 10^−5^**	**1.58 × 10^−3^**
Non-motor actin binding protein (PC00165)	12.57	4.56 × 10^−7^	8.80 × 10^−5^
Isomerase (PC00135)	10.37	3.63 × 10^−3^	4.67 × 10^−2^
**MOLECULAR FUNCTION**			
Dihydropyrimidinase activity (GO:0004157)	69.11	2.96 × 10^−5^	2.73 × 10^−3^
Hydrolase activity, acting on carbon-nitrogen (but not peptide) bonds, in cyclic amides (GO:0016812)	51.83	5.74 × 10^−5^	3.54 × 10^−3^
Clathrin binding (GO:0030276)	11.52	5.29 × 10^−4^	1.28 × 10^−2^
**Unfolded protein binding (GO:0051082)**	**10.05**	**8.56 × 10^−4^**	**1.58 × 10^−2^**
**BIOLOGICAL PROCESS**			
Pyrimidine nucleobase catabolic process (GO:0006208)	59.24	4.20 × 10^−5^	9.16 × 10^−3^
Pyrimidine nucleobase catabolic process (GO:0006208)	59.24	4.20 × 10^−5^	9.16 × 10^−3^
Pyrimidine nucleobase metabolic process (GO:0006206)	37.7	1.25 × 10^−4^	1.51 × 10^−2^
Pyrimidine nucleobase metabolic process (GO:0006206)	37.7	1.25 × 10^−4^	1.51 × 10^−2^
**Protein stabilization (GO:0050821)**	**29.1**	**2.04 × 10^−5^**	**1.48 × 10^−2^**
Regulation of protein stability (GO:0031647)	27.64	2.43 × 10^−5^	1.32 × 10^−2^
Nucleobase metabolic process (GO:0009112)	24.04	3.95 × 10^−5^	9.56 × 10^−3^
Pyrimidine-containing compound metabolic process (GO:0072527)	18.85	7.44 × 10^−4^	3.96 × 10^−2^
Establishment or maintenance of cell polarity (GO:0007163)	12.8	6.55 × 10^−5^	1.02 × 10^−2^

† For complete information of GO analysis performed by GeneOntology Panther see Supplementary Materials Table S3. Table shows data filtered by Fold Enrichment > 10. FDR (False Discovery Rate).

**Table 2:** Reactome pathways. Table shows pathways in which proteins identified as chaperones in STRING diagram are involved (*p* value < 0.05).

Identifier	Pathway Name	#Found	#Total	*p* Value	FDR	Submitted Entities Found
R-HSA-422475	Axon guidance	21	558	1.80 × 10^−5^	0.0056	AP2A2, HSP90AA1, DAG1, CLASP2, MAP2K1, DPYSL5, AP2A1, EZR, PRKACA, RPL35, CSNK2A1, PLXNA4, DPYSL4, DLG3, DLG4, CRMP1, MSN, RPS25, HSP90AB1, PFN2, RPLP2
R-HSA-69275	G2/M Transition	11	198	7.85 × 10^−5^	0.0065	PRKACA, DCTN1, HSP90AA1, PPP2R2A, CKAP5, PPME1, SKP1, PAFAH1B1, PPP2R1A, HSP90AB1, PRKAR2B
R-HSA-3371568	Attenuation phase	3	14	9.22 × 10^−4^	0.0350	HSP90AA1, HSP90AB1, FKBP4
R-HSA-399954	Sema3A PAK dependent Axon repulsion	3	16	0.00135	0.0459	HSP90AA1, PLXNA4, HSP90AB1
R-HSA-9613829	Chaperone Mediated Autophagy	3	22	0.00331	0.0744	HSP90AA1, HSP90AB1, GFAP
R-HSA-168249	Innate Immune System	27	1187	0.00399	0.0744	HMGB1, HSP90AA1, S100A1, PPIA, PRDX6, FABP5, C4B, C4B_2, SUGT1, BAIAP2, SKP1, HEBP2, GSTP1, GPI, AP2A2, PIN1, COTL1, HSP90B1, MGST1, PPP2R1A, MAP2K1, PADI2, MAPK10, UBE2M, PRKACA, HMOX2, CD44, HSP90AB1
R-HSA-5336415	Uptake and function of diphtheria toxin	2	7	0.00405	0.0744	HSP90AA1, HSP90AB1
R-HSA-5339562	Uptake and actions of bacterial toxins	4	48	0.00407	0.0744	HSP90AA1, PDCD6IP, MAP2K1, HSP90AB1
R-HSA-3371571	HSF1-dependent transactivation	3	24	0.00421	0.0744	P07900, P08238, Q02790
R-HSA-3371497	HSP90 chaperone cycle for steroid hormone receptors (SHR)	4	57	0.00921	0.0760	DCTN1, HSP90AA1, HSP90AB1, FKBP4
R-HSA-3371556	Cellular response to heat stress	5	95	0.01141	0.0799	HSP90AA1, HSPA4L, BAG3, HSP90AB1, FKBP4
R-HSA-3371511	HSF1 activation	2	12	0.04212	0.1685	HSP90AA1, HSP90AB1
R-HSA-449147	Signaling by Interleukins	11	456	0.04615	0.1728	HMGB1, PRKACA, HSP90AA1, PPIA, FSCN1, HSP90B1, SKP1, PPP2R1A, MAP2K1, MSN, MAPK10
R-HSA-8953897	Cellular responses to external stimuli	13	579	1.80 × 10^−5^	0.0056	CSRP1, DCTN1, HSP90AA1, HSPA4L, PRDX6, MAPK10, FKBP4, RPL35, BAG3, GSTP1, RPS25, HSP90AB1, RPLP2

**Table 3:** Human samples. Detailed information about the samples employed in the study.

Case	Gender	Age (y)	PMD (h)	Brain Weight (g)	Cause of Death	Braak Stage	Treatment	Assay
AD cases (*n* = 16)								
1	F	74	4:00	1042	Cardiorespiratory arrest	V	Formalin-fixed	IHC, IFC_1_
2	F	80	4:00	910	Respiratory infection	V	Formalin-fixed	IHC, IFC_1_, IFC_2_
3	M	77	5:00	1330	Sepsis (respiratory origin)	VI	Formalin-fixed	IHC, IFC_1_
4	F	84	2:00	920	Unknow	V	Formalin-fixed	IHC, IFC_1_
5	M	77	6:00	1060	Acute respiratory infection	VI	Formalin-fixed	IHC, IFC_1_
6	M	92	6:00	960	Unknow	VI	Formalin-fixed	IHC, IFC_2_
7	M	75	3:00	1050	Multiorganic arrest	V	Formalin-fixed	IHC
8	F	85	2:00	1150	Cardiorespiratory arrest	VI	Formalin-fixed	IHC, IFC_2_
9	F	83	2:00	1000	Respiratory insufficiency	VI	Formalin-fixed	IHC
10	F	81	6:30	935	Cardiorespiratory arrest	VI	Fresh-frozen	PR, WB
11	F	75	16:15	1200	Septic shock, sacral ulcer	VI	Fresh-frozen	PR, WB
12	M	80	21:45	1300	Respiratory insufficiency	VI (LBD)	Fresh-frozen	PR, WB
13	F	80	5:00	1060	Acute heart failure	VI	Fresh-frozen	PR, WB
14	F	72	14:00	1060	Cardiorespiratory arrest, Deep venous thrombosis	VI (LBD)	Fresh-frozen	PR, WB
15	F	90	12:15	920	Respiratory insufficiency, respiratory infection	VI	Fresh-frozen	PR
16	F	76	11:10	900	Respiratory insufficiency	VI	Fresh-frozen	WB
Non-AD cases (*n* = 16)								
17	M	84	3:00	1400	Non filiate miocardiopathy/ Cardiac arrest	-	Formalin-fixed	IHC, IFC_2_
18	F	81	5:00	1100	Pionefritis by E. coli/ Multiorganic arrest	-	Formalin-fixed	IHC
19	M	88	3:00	1285	Unknow	II	Formalin-fixed	IHC
20	M	83	4:00	1152	Unknow	II	Formalin-fixed	IHC
21	F	62	2:00	1050	Myelodysplastic Syndrome	-	Formalin-fixed	IHC, IFC_2_
22	M	63	2:00	1400	Breast adenocarcinoma	-	Formalin-fixed	IHC, IFC_2_
23	M	58	6:00	1500	Acute myocardial infarction	-	Formalin-fixed	IHC
24	M	53	5:00	1300	Rectum adenocarcinoma	-	Formalin-fixed	IHC
25	M	78	4:00	1100	Lung carcinoma	-	Formalin-fixed	IHC
26	F	83	7:20	1320	Intestinal embolism, surgery ischemia	II	Fresh-frozen	PR, WB
27	M	83	13:00	1630	AgD I, pathology due to argyrophile granules/cardiorespiratory insufficiency	-	Fresh-frozen	PR
28	F	37	9:00	1200	Refractory septic shock	-	Fresh-frozen	PR, WB
29	M	57	12:00	1560	Lung Carcinoma	-	Fresh-frozen	PR
30	M	68	4:10	1350	Sepsis		Fresh-frozen	WB
31	F	82	4:00	800	Respiratory insufficiency	-	Fresh-frozen	WB
32	F	71	7:08	975	Cardiorespiratory arrest	-	Fresh-frozen	WB

F (female), M (male), PMD (Postmortem Delay), LBD (Lewy Body Dementia), IHC (immunohistochemistry used for stereological estimations), IF1 (immunofluorescence against GFAP and pathological proteins), IF2 (immunofluorescence against selected proteins of proteomic analysis), PR (proteomic analysis), WB (Western blot analysis).

## Data Availability

All data generated or analyzed during this study are included in this published article (and its Supplementary Materials).

## Supplementary Materials

The following are available online at https://www.mdpi.com/article/10.3390/ijms23010165/s1.
