# Supplementary material for: Neurodegeneration and Astrogliosis in the Human CA1 Hippocampal Subfield Are Related to hsp90ab1 and bag3 in Alzheimer’s Disease
Source: Int J Mol Sci. 2021 Dec 23;23(1):165. doi: 10.3390/ijms23010165 (PMC8745315; doi:10.3390/ijms23010165)
Supplement: Supplementary file 1 [file ijms-23-00165-s001.zip › File S1.pdf]

Hippocampus Cavalieri Data

Case	Volume Corrected for OverProjection (mm³)	Coefficient of Error (Gundersen). m=1	Section Cut Thickness (µm)	Section Evaluation Interval	Grid Size (µm)	Sections	Count
**AD cases**							
**1**	152.378	0.024	50	13	250	4	3831
**2**	121.925	0.021	50	13	250	4	3069
**3**	76.1	0.021	50	13	250	4	1914
**4**	69.9344	0.030	50	13	250	4	1767
**5**	117.869	0.020	50	13	250	4	2963
**6**	84.0125	0.023	50	13	250	4	2111
**7**	99.4031	0.023	50	13	250	4	2497
**8**	87.6937	0.023	50	13	250	4	2202
**9**	63.2687	0.024	50	13	250	4	1590
**Non-AD cases**							
**16**	246.991	0.023	50	13	250	4	6209
**17**	188.597	0.022	50	13	250	4	4738
**18**	112.791	0.023	50	13	250	4	2833
**19**	101.65	0.022	50	13	250	4	2554
**20**	145.113	0.021	50	13	250	4	3646
**21**	101.869	0.023	50	13	250	4	2560
**22**	137.644	0.031	50	13	250	4	3466
**23**	132.084	0.019	50	13	250	4	3323
**24**	133.553	0.023	50	13	250	4	3354

Dentate Gyrus Cavalieri Data

Case	Volume Corrected for OverProjection (mm³)	Coefficient of Error (Gundersen). m=1	Section Cut Thickness (µm)	Section Evaluation Interval	Grid Size (µm)	Sections	Count
**AD cases**							
**1**	41.675	0.035	50	13	250	4	1061
**2**	47.0437	0.022	50	13	250	4	1186
**3**	19.8562	0.025	50	13	250	4	499
**4**	17.7313	0.023	50	13	250	4	446
**5**	40.8906	0.030	50	13	250	4	1035
**6**	27.9531	0.024	50	13	250	4	704
**7**	31.3969	0.023	50	13	250	4	789
**8**	25.2813	0.022	50	13	250	4	635
**9**	26.6156	0.024	50	13	250	4	669
**Non-AD cases**							
**16**	84.725	0.021	50	13	250	4	2136
**17**	68.7406	0.021	50	13	250	4	1729
**18**	30.0938	0.027	50	13	250	4	758
**19**	24.5844	0.024	50	13	250	4	618
**20**	34.7687	0.024	50	13	250	4	873
**21**	23.5281	0.025	50	13	250	4	591
**22**	32.3812	0.024	50	13	250	4	815
**23**	40.7125	0.020	50	13	250	4	1026
**24**	36.9906	0.023	50	13	250	4	930

CA1 Cavalieri Data

Case	Volume Corrected for OverProjection (mm³)	Coefficient of Error (Gundersen). m=1	Section Cut Thickness (µm)	Section Evaluation Interval	Grid Size (µm)	Sections	Count
**AD cases**							
**1**	54.5812	0.025	50	13	250	4	1372
**2**	35.9937	0.024	50	13	250	4	904
**3**	23.6594	0.024	50	13	250	4	595
**4**	26.9656	0.026	50	13	250	4	679
**5**	41.0156	0.023	50	13	250	4	1032
**6**	33.3688	0.022	50	13	250	4	839
**7**	47.0969	0.022	50	13	250	4	1183
**8**	44.8281	0.023	50	13	250	4	1126
**9**	24.3969	0.027	50	13	250	4	614
**Non-AD cases**							
**16**	82.4688	0.026	50	13	250	4	2074
**17**	70.9094	0.039	50	13	250	4	1729
**18**	59.3937	0.023	50	13	250	4	1493
**19**	60.4469	0.022	50	13	250	4	1519
**20**	88.3906	0.020	50	13	250	4	2222
**21**	61.4156	0.023	50	13	250	4	1544
**22**	87.575	0.035	50	13	250	4	2209
**23**	85.0938	0.025	50	13	250	4	2147
**24**	68.5156	0.023	50	13	250	4	1722

CA2 Cavalieri Data

Case	Volume Corrected for OverProjection (mm³)	Coefficient of Error (Gundersen). m=1	Section Cut Thickness (µm)	Section Evaluation Interval	Grid Size (µm)	Sections	Count
**AD cases**							
**1**	25.5938	0.023	50	13	250	4	645
**2**	22.3281	0.025	50	13	250	4	564
**3**	13.1687	0.029	50	13	250	4	335
**4**	9.11875	0.060	50	13	250	4	235
**5**	17.0375	0.020	50	13	250	4	430
**6**	6	0.034	50	13	250	4	151
**7**	5.84375	0.031	50	13	250	4	147
**8**	7.35	0.034	50	13	250	4	185
**9**	4.07813	0.033	50	13	250	4	103
**Non-AD cases**							
**16**	52.5188	0.030	50	13	250	4	1327
**17**	34.9	0.023	50	13	250	4	882
**18**	8.22188	0.026	50	13	250	4	207
**19**	5.04062	0.030	50	13	250	4	127
**20**	10.2125	0.024	50	13	250	4	257
**21**	8.31875	0.029	50	13	250	4	209
**22**	8.66563	0.028	50	13	250	4	218
**23**	6.35313	0.028	50	13	250	4	160
**24**	11.5156	0.031	50	13	250	4	291

CA3 Cavalieri Data

Case	Volume Corrected for OverProjection (mm³)	Coefficient of Error (Gundersen). m=1	Section Cut Thickness (μm)	Section Evaluation Interval	Grid Size (μm)	Sections	Count
**AD cases**							
**1**	24.4156	0.051	50	13	250	4	630
**2**	13.75	0.030	50	13	250	4	348
**3**	17.4406	0.020	50	13	250	4	439
**4**	14.1906	0.045	50	13	250	4	363
**5**	16.5031	0.031	50	13	250	4	419
**6**	14.7188	0.035	50	13	250	4	373
**7**	12.4188	0.027	50	13	250	4	213
**8**	8.73438	0.026	50	13	250	4	220
**9**	6.52187	0.028	50	13	250	4	464
**Non-AD cases**							
**16**	22.2031	0.039	50	13	250	4	568
**17**	8.475	0.036	50	13	250	4	215
**18**	12.6938	0.024	50	13	250	4	319
**19**	9.41875	0.024	50	13	250	4	237
**20**	9.03438	0.026	50	13	250	4	227
**21**	6.00312	0.031	50	13	250	4	151
**22**	6.61562	0.033	50	13	250	4	167
**23**	8.65	0.028	50	13	250	4	218
**24**	13.7937	0.024	50	13	250	4	347

DG NeuN stereological quantification data

Case	Total Markers Counted	Number of Sections	Number of Sampling Sites	Coefficient of Error (Gundersen). m=1	Counting Frame Area (XY) (μm²)	Sampling Grid Area (XY) (μm²)	Estimated Population using Mean Section Thickness with Counts	Measured Volume (mm³)	Numerical Density (cell/mm³)
**AD cases**									
**1**	391	4	235	0.06	2500	250000	786629.31	35.4673	22179.00178
**2**	654	4	279	0.05	2500	122500	1216460.63	43.5345	27942.45093
**3**	232	4	132	0.08	2500	250000	449272.53	20.4099	22012.48071
**4**	297	4	135	0.07	2500	250000	535593.13	21.4682	24948.20851
**5**	288	4	171	0.06	2500	250000	590008.63	26.4294	22323.95098
**6**	496	4	198	0.05	2500	250000	887326.38	30.7507	28855.48557
**7**	548	4	210	0.05	2500	250000	1066441.25	32.5432	32770.01801
**8**	328	4	145	0.06	2500	250000	645082.63	22.5	28670.33911
**9**	564	4	160	0.05	2500	250000	964080.69	25.8675	37269.95999
**Non-AD cases**									
**16**	727	4	566	0.04	2500	250000	1530572.5	89.9573	17014.43351
**17**	952	4	402	0.04	2500	250000	1925861.88	61.6978	31214.43358
**18**	375	4	153	0.06	2500	250000	886092	23.5368	37647.08881
**19**	407	4	121	0.06	2500	250000	1053389.88	19.1076	55129.36633
**20**	420	4	186	0.06	2500	250000	818470.88	28.4788	28739.65476
**21**	239	4	136	0.07	2500	250000	476246.38	20.353	23399.32098
**22**	514	4	198	0.05	2500	250000	1025459.81	30.7091	33392.70151
**23**	385	4	225	0.06	2500	250000	819446.25	35.2065	23275.42499
**24**	540	4	236	0.05	2500	250000	1087637.75	37.1543	29273.53631

CA1 NeuN stereological quantification data

Case	Total Markers Counted	Number of Sections	Number of Sampling Sites	Coefficient of Error (Gundersen). m=1	Counting Frame Area (XY) (µm²)	Sampling Grid Area (XY) (µm²)	Estimated Population using Mean Section Thickness with Counts	Measured Volume (mm³)	Numerical Density (cell/mm³)
**AD cases**									
**1**	367	4	483	0.06	2500	122500	399333.28	36.7465	10867.24668
**2**	334	4	416	0.06	2500	122500	310032.84	31.3596	9886.377377
**3**	147	4	304	0.09	2500	122500	128358.04	22.5981	5680.036817
**4**	168	4	285	0.08	2500	122500	153191.41	21.3731	7167.486701
**5**	147	4	296	0.08	2500	122500	146848.98	22.0478	6660.482225
**6**	99	4	381	0.1	2500	122500	87557.04	29.0264	3016.462255
**7**	225	4	479	0.07	2500	122500	234498.42	36.5138	6422.186132
**8**	146	4	468	0.09	2500	122500	159315.84	35.9	4437.767131
**9**	195	4	452	0.08	2500	62500	90618.70	17.2769	5245.078689
**Non-AD cases**									
**16**	283	4	626	0.07	2500	122500	312730.06	47.3804	6600.409874
**17**	361	4	769	0.06	2500	122500	382628.53	58.1302	6582.267565
**18**	358	4	513	0.06	2500	122500	415344.5	39.2913	10570.90246
**19**	177	4	426	0.08	2500	122500	205755.89	32.625	6306.693946
**20**	618	4	903	0.05	2500	122500	595535.75	70.1547	8488.893118
**21**	476	4	699	0.05	2500	122500	477105.75	54.1433	8811.907475
**22**	576	4	1005	0.05	2500	122500	595293	77.2162	7709.431441
**23**	364	4	607	0.06	2500	122500	421029.94	46.3443	9084.826829
**24**	275	4	644	0.07	2500	122500	255604.88	48.7782	5240.145803

CA2 NeuN stereological quantification data

Case	Total Markers Counted	Number of Sections	Number of Sampling Sites	Coefficient of Error (Gundersen). m=1	Counting Frame Area (XY) (µm²)	Sampling Grid Area (XY) (µm²)	Estimated Population using Mean Section Thickness with Counts	Measured Volume (mm³)	Numerical Density (cell/mm³)
**AD cases**									
**1**	955	4	1577	0.04	2500	22500	183543.19	21.7365	8444.008465
**2**	924	4	1368	0.04	2500	22500	173014.80	18.8843	9161.832845
**3**	287	4	519	0.07	2500	22500	52978.95	7.06552	7584.784984
**4**	476	4	620	0.06	2500	22500	85853.50	8.508	10090.91443
**5**	469	4	911	0.05	2500	22500	93391.33	12.5449	7211.767332
**6**	212	4	406	0.07	2500	22500	37910.25	5.44984	6956.213393
**7**	206	4	415	0.07	2500	22500	46689.45	5.6072	8326.696034
**8**	237	4	596	0.06	2500	22500	54374.19	8.1884	6267.584632
**9**	203	4	334	0.07	2500	22500	34860.35	4.36278	7990.398324
**Non-AD cases**									
**16**	996	4	1951	0.04	2500	22500	223143.91	27.2396	8191.893787
**17**	884	4	1858	0.04	2500	22500	173739.19	25.9582	6693.036882
**18**	260	4	355	0.07	2500	22500	62291.13	4.69638	13263.64775
**19**	151	4	316	0.09	2500	22500	38482.58	4.17371	9220.233318
**20**	366	4	563	0.06	2500	22500	71674.88	7.64981	9369.498066
**21**	204	4	466	0.08	2500	22500	39230.67	6.27401	6252.886113
**22**	219	4	488	0.07	2500	22500	44855.15	6.55061	6847.4768
**23**	234	4	379	0.07	2500	22500	51211.37	5.09704	10047.27646
**24**	217	4	527	0.07	2500	22500	173739.19	25.9582	6693.036882

CA3 NeuN stereological quantification data

Case	Total Markers Counted	Number of Sections	Number of Sampling Sites	Coefficient of Error (Gundersen). m=1	Counting Frame Area (XY) (μm²)	Sampling Grid Area (XY) (μm²)	Estimated Population using Mean Section Thickness with Counts	Measured Volume (mm³)	Numerical Density (cell/mm³)
**AD cases**									
**1**	1599	4	1765	0.05	2500	22500	294057.19	24.8134	11850.74153
**2**	1045	4	1039	0.04	2500	22500	183199.33	14.2153	12887.47547
**3**	754	4	1170	0.05	2500	22500	141353.30	16.2167	8681.891507
**4**	1264	4	1480	0.04	2500	22500	217387.78	20.6977	10502.99212
**5**	781	4	1246	0.05	2500	22500	148254.98	17.2781	8719.490569
**6**	427	4	862	0.06	2500	22500	79807.47	11.8087	6758.362055
**7**	364	4	592	0.06	2500	22500	82213.02	8.18785	10040.85566
**8**	327	4	630	0.06	2500	22500	68477.55	8.52598	8305.282208
**9**	355	4	500	0.06	2500	22500	58619.00	6.78565	8638.671314
**Non-AD cases**									
**16**	629	4	1072	0.07	2500	22500	125169.2	14.9661	8363.514877
**17**	518	4	812	0.06	2500	22500	98042.73	11.3217	8659.718064
**18**	388	4	555	0.06	2500	22500	92957.53	7.3819	12592.62927
**19**	281	4	390	0.06	2500	22500	70998.33	5.32111	13342.76683
**20**	562	4	738	0.05	2500	22500	106964.33	10.1751	10512.36155
**21**	308	4	482	0.06	2500	22500	61077.09	6.60425	9248.149298
**22**	291	4	493	0.07	2500	22500	55489.28	6.61889	8383.472153
**23**	252	4	488	0.07	2500	22500	53992.43	6.57666	8209.70371
**24**	395	4	719	0.06	2500	22500	98042.73	11.3217	8659.718064

DG Iba-1 stereological quantification data

Case	Total Markers Counted	Number of Sections	Number of Sampling Sites	Coefficient of Error (Gundersen). m=1	Counting Frame Area (XY) (µm²)	Sampling Grid Area (XY) (µm²)	Estimated Population using Mean Section Thickness with Counts	Measured Volume (mm³)	Numerical Density (cell/mm³)
**AD cases**									
**1**	212	4	236	0.07	2500	250000	389297.94	36.4299	10686.22038
**2**	570	4	267	0.05	2500	250000	1034321.63	41.4721	24940.17978
**3**	290	4	118	0.07	2500	250000	480105.75	18.9704	25308.15112
**4**	333	4	152	0.06	2500	250000	567029.19	23.2779	24359.12131
**5**	221	4	198	0.07	2500	250000	368768.75	31.1065	11855.03834
**6**	135	4	325	0.09	2500	122500	129286.54	25.2124	5127.895004
**7**	289	4	168	0.06	2500	250000	614481.81	25.1463	24436.27134
**8**	248	4	129	0.06	2500	250000	426804.28	19.842	21510.14414
**9**	311	4	165	0.06	2500	250000	526696.25	25.7378	20463.91883
**Non-AD cases**									
**16**	319	4	489	0.07	2500	250000	686279.94	76.2462	9000.841222
**17**	689	4	375	0.04	2500	250000	1332739.25	58.8152	22659.77587
**18**	232	4	138	0.07	2500	250000	577360.5	20.5484	28097.58911
**19**	198	4	136	0.08	2500	250000	372387.25	20.7867	17914.68824
**20**	325	4	190	0.06	2500	250000	600533.63	30.0927	19956.12325
**21**	103	4	224	0.1	2500	160000	131189.58	22.0119	5959.938942
**22**	102	4	241	0.1	2500	160000	139682.11	23.143	6035.609472
**23**	176	4	197	0.08	2500	250000	404964	30.1632	13425.76384
**24**	299	4	195	0.06	2500	250000	636878.81	30.1547	21120.38289

CA1 Iba-1 stereological quantification data

Case	Total Markers Counted	Number of Sections	Number of Sampling Sites	Coefficient of Error (Gundersen). m=1	Counting Frame Area (XY) (μm²)	Sampling Grid Area (XY) (μm²)	Estimated Population using Mean Section Thickness with Counts	Measured Volume (mm³)	Numerical Density (cell/mm³)
**AD cases**									
**1**	541	4	270	0.05	2500	250000	1046358.5	42.1285	24837.30729
**2**	363	4	201	0.06	2500	250000	656989.75	30.564	21495.54214
**3**	395	4	126	0.05	2500	250000	653937.19	20.1346	32478.28067
**4**	268	4	148	0.07	2500	250000	653937.19	20.1346	32478.28067
**5**	281	4	160	0.06	2500	250000	468886.91	23.9886	19546.23905
**6**	118	4	141	0.09	2500	250000	214237.73	20.887	10256.98904
**7**	254	4	178	0.07	2500	250000	591909	28.1619	21018.07762
**8**	314	4	201	0.06	2500	250000	540389.25	31.1253	17361.73627
**9**	217	4	120	0.07	2500	250000	407477.78	18.2874	22281.88698
**Non-AD cases**									
**16**	260	4	327	0.07	2500	250000	559350.44	49.8417	11222.53936
**17**	292	4	340	0.07	2500	250000	591256.44	54.1695	10914.93257
**18**	261	4	271	0.07	2500	250000	642565.75	42.2469	15209.77279
**19**	251	4	288	0.07	2500	250000	472066.72	44.4192	10627.53764
**20**	358	4	373	0.06	2500	250000	666085.06	58.0572	11472.91051
**21**	171	4	296	0.08	2500	250000	348141.16	45.6045	7633.921214
**22**	170	4	351	0.09	2500	250000	367208.63	55.8872	6570.531893
**23**	113	4	287	0.1	2500	250000	270540.91	45.4322	5954.827413
**24**	225	4	272	0.07	2500	250000	500642.09	42.1003	11891.65137

CA2 Iba-1 stereological quantification data

Case	Total Markers Counted	Number of Sections	Number of Sampling Sites	Coefficient of Error (Gundersen). m=1	Counting Frame Area (XY) (μm²)	Sampling Grid Area (XY) (μm²)	Estimated Population using Mean Section Thickness with Counts	Measured Volume (mm³)	Numerical Density (cell/mm³)
**AD cases**									
**1**	668	4	464	0.04	2500	62500	309857.69	17.6593	17546.43106
**2**	711	4	422	0.05	2500	62500	332681.19	16.2316	20495.89628
**3**	633	4	266	0.05	2500	62500	272288.31	10.2755	26498.78935
**4**	357	4	207	0.08	2500	62500	162671.27	7.73013	21043.79487
**5**	462	4	272	0.06	2500	62500	208757.56	10.2178	20430.77375
**6**	230	4	155	0.07	2500	62500	116537.79	5.61157	20767.41268
**7**	154	4	114	0.09	2500	62500	92782.77	4.27876	21684.49971
**8**	319	4	181	0.06	2500	62500	146337.02	6.71713	21785.64655
**9**	161	4	98	0.08	2500	62500	82013.29	3.64962	22471.73404
**Non-AD cases**									
**16**	737	4	808	0.05	2500	62500	432021.88	31.3056	13800.14694
**17**	736	4	579	0.04	2500	62500	395426.25	22.3274	17710.35812
**18**	144	4	142	0.09	2500	62500	95609.07	5.39671	17716.17708
**19**	174	4	159	0.08	2500	62500	93820.61	6.15084	15253.30036
**20**	223	4	204	0.07	2500	62500	109888.76	7.44368	14762.69265
**21**	172	4	442	0.08	2500	22500	33706.89	5.98362	5633.193619
**22**	136	4	162	0.09	2500	62500	77659.02	6.24686	12431.68888
**23**	178	4	370	0.08	2500	22500	37052.72	4.86664	7613.614321
**24**	122	4	153	0.10	2500	62500	72975.63	5.83281	12511.23044

CA3 Iba-1 stereological quantification data

Case	Total Markers Counted	Number of Sections	Number of Sampling Sites	Coefficient of Error (Gundersen). m=1	Counting Frame Area (XY) (μm²)	Sampling Grid Area (XY) (μm²)	Estimated Population using Mean Section Thickness with Counts	Measured Volume (mm³)	Numerical Density (cell/mm³)
**AD cases**									
**1**	473	4	595	0.06	2500	62500	226688.22	23.1233	9803.454524
**2**	510	4	300	0.04	2500	62500	229855.28	11.1966	20529.02488
**3**	887	4	405	0.04	2500	62500	381547.75	15.6861	24323.93967
**4**	962	4	544	0.04	2500	62500	422394.84	21.1992	19925.03679
**5**	319	4	344	0.07	2500	62500	144142.13	13.2798	10854.23952
**6**	200	4	283	0.08	2500	62500	97683.82	10.8925	8967.988983
**7**	233	4	193	0.08	2500	62500	141196.41	7.4218	19024.55065
**8**	291	4	195	0.06	2500	62500	133492.38	7.32145	18233.0522
**9**	285	4	154	0.06	2500	62500	143236.81	5.78606	24755.50029
**Non-AD cases**									
**16**	341	4	595	0.07	2500	62500	199890.7	19.0489	10493.55606
**17**	287	4	247	0.08	2500	62500	135100.69	9.02546	14968.84259
**18**	288	4	230	0.06	2500	62500	196129.38	8.6363	22709.88502
**19**	220	4	196	0.07	2500	62500	118623.74	7.24738	16367.81016
**20**	318	4	252	0.06	2500	62500	151605.06	9.66001	15694.08934
**21**	168	4	463	0.08	2500	22500	31847.67	6.21482	5124.471827
**22**	135	4	440	0.09	2500	22500	26973.71	5.88121	4586.421842
**23**	189	4	475	0.08	2500	22500	39346.64	6.3264	6219.436014
**24**	242	4	231	0.07	2500	62500	137996.86	8.91189	15484.57847

DG GFAP stereological quantification data

Case	Total Markers Counted	Number of Sections	Number of Sampling Sites	Coefficient of Error (Gundersen). m=1	Counting Frame Area (XY) (µm²)	Sampling Grid Area (XY) (µm²)	Estimated Population using Mean Section Thickness with Counts	Measured Volume (mm³)	Numerical Density (cell/mm³)
**AD cases**									
**1**	262	4	181	0.08	2500	250000	488276.91	27.9982	17439.58219
**2**	-	-	-	-	-	-	-	-	-
**3**	156	4	116	0.09	2500	250000	295295.22	17.8509	16542.31551
**4**	182	4	98	0.08	2500	250000	343115.88	15.3836	22304.00426
**5**	175	4	175	0.08	2500	250000	330501.16	28.114	11755.75016
**6**	237	4	149	0.07	2500	250000	430934.72	23.0958	18658.57515
**7**	161	4	156	0.08	2500	250000	350292.72	23.8874	14664.33015
**8**	231	4	156	0.07	2500	250000	425061.5	23.9615	17739.35271
**9**	250	4	167	0.07	2500	250000	412818.41	25.5512	16156.5175
**Non-AD cases**									
**16**	274	4	377	0.07	2500	250000	489327.47	60.1315	8137.622877
**17**	310	4	331	0.06	2500	250000	549988	49.4062	11131.96319
**18**	148	4	131	0.08	2500	250000	330101.34	20.4316	16156.41164
**19**	147	4	117	0.09	2500	250000	314195.91	18.9723	16560.7707
**20**	181	4	226	0.08	2500	250000	323703.72	35.3578	9155.086572
**21**	169	4	129	0.08	2500	250000	333490.34	20.6463	16152.54743
**22**	174	4	171	0.08	2500	250000	338898.47	25.6874	13193.17915
**23**	184	4	167	0.08	2500	250000	416264.81	26.4868	15715.93435
**24**	212	4	190	0.07	2500	250000	469839.81	29.5165	15917.87

CA1 GFAP stereological quantification data

Case		Total Markers Counted	Number of Sections	Number of Sampling Sites	Coefficient of Error (Gundersen). m=1	Counting Frame Area (XY) (μm^2)	Sampling Grid Area (XY) (μm^2)	Estimated Population using Mean Section Thickness with Counts	Measured Volume (mm^3)	Numerical Density ($cell/mm^3$)
	AD cases									
**1**		541	4	912	0.05	2500	90000	386385.94	51.5566	7494.403044
**2**		-	-	-	-	-	-	-	-	-
**3**		342	4	417	0.06	2500	90000	236610	22.8612	10349.8504
**4**		431	4	470	0.05	2500	90000	166867.72	29.9485	5571.822295
**5**		233	4	539	0.07	2500	90000	166867.72	29.9485	5571.822295
**6**		414	4	416	0.06	2500	90000	260734.55	31.1018	8383.262384
**7**		324	4	588	0.06	2500	90000	260734.55	31.1018	8383.262384
**8**		566	4	677	0.05	2500	90000	386244.41	37.6545	10257.58966
**9**		386	4	388	0.06	2500	90000	236610	22.8612	10349.8504
	Non-AD cases									
**16**		179	4	992	0.09	2500	90000	126051.77	55.9904	2251.310403
**17**		228	4	774	0.07	2500	90000	158084.19	43.0886	3668.817042
**18**		186	4	759	0.08	2500	90000	158085.75	42.5016	3719.524677
**19**		215	4	690	0.07	2500	90000	170127.72	38.6278	4404.28189
**20**		204	4	1107	0.07	2500	90000	135798.78	62.8412	2160.98324
**21**		283	4	814	0.06	2500	90000	215318.55	45.1563	4768.294789
**22**		216	4	1300	0.07	2500	90000	161681.14	73.3751	2203.487832
**23**		521	4	873	0.05	2500	90000	434617.47	49.6593	8751.985429
**24**		290	4	800	0.06	2500	90000	242778.34	44.6774	5434.030181

CA2 GFAP stereological quantification data

Case	Total Markers Counted	Numbe r of Sections	Number of Sampling Sites	Coefficient of Error (Gundersen). m=1	Counting Frame Area (XY) (μm²)	Sampling Grid Area (XY) (μm²)	Estimated Population using Mean Section Thickness with Counts	Measured Volume (mm³)	Numerical Density (cell/mm³)
**AD cases**									
**1**	723	4	1104	0.05	2500	22500	127412.98	15.3456	8302.899854
**2**	-	-	-	-	-	-	-	-	-
**3**	418	4	670	0.06	2500	22500	73339.25	9.06414	8091.142679
**4**	633	4	607	0.06	2500	22500	104311.66	18.46	5650.685807
**5**	212	4	476	0.07	2500	22500	104311.66	18.46	5650.685807
**6**	412	4	446	0.06	2500	22500	67525.74	5.90275	11439.70861
**7**	372	4	389	0.05	2500	22500	81355.41	5.24604	15507.96601
**8**	418	4	519	0.06	2500	22500	77319.67	6.96592	11099.70686
**9**	245	4	321	0.07	2500	22500	73339.25	9.06414	8091.142679
**Non-AD cases**									
**16**	231	4	709	0.07	2500	62500	112241.76	27.5731	4070.69789
**17**	1144	4	1839	0.03	2500	22500	195551.7	25.6889	7612.303368
**18**	257	4	409	0.07	2500	22500	54709.8	5.53548	9883.478939
**19**	250	4	402	0.07	2500	22500	50545.29	5.40665	9348.726106
**20**	193	4	408	0.08	2500	22500	32450.56	5.48751	5913.530909
**21**	237	4	380	0.07	2500	22500	43081.7	5.07974	8481.083678
**22**	341	4	462	0.06	2500	22500	32450.56	5.48751	5913.530909
**23**	397	4	331	0.05	2500	22500	85982.95	4.35008	19765.83189
**24**	308	4	497	0.07	2500	22500	195551.7	25.6889	7612.303368

CA3 GFAP stereological quantification data

Case	Total Markers Counted	Number of Sections	Number of Sampling Sites	Coefficient of Error (Gundersen). m=1	Counting Frame Area (XY) (μm²)	Sampling Grid Area (XY) (μm²)	Estimated Population using Mean Section Thickness with Counts	Measured Volume (mm³)	Numerical Density (cell/mm³)
**AD cases**									
**1**	318	4	417	0.07	2500	62500	151739.8	15.9804	9495.369327
**2**	-	-	-	-	-	-	-	-	-
**3**	401	4	452	0.07	2500	62500	193234.02	17.4451	11076.69317
**4**	563	4	444	0.05	2500	62500	138479.52	16.9796	8155.640887
**5**	293	4	440	0.07	2500	62500	138479.52	16.9796	8155.640887
**6**	540	4	389	0.05	2500	62500	139435.06	8.98266	15522.6915
**7**	245	4	237	0.07	2500	62500	139435.06	8.98266	15522.6915
**8**	173	4	217	0.08	2500	62500	86211.67	8.2389	10463.9782
**9**	168	4	165	0.08	2500	62500	193234.02	17.4451	11076.69317
**Non-AD cases**									
**16**	237	4	488	0.08	2500	62500	106844.99	18.7634	5694.329919
**17**	345	4	395	0.06	2500	62500	159339.42	14.8687	10716.43251
**18**	169	4	187	0.08	2500	62500	100381.72	6.95399	14435.12573
**19**	144	4	143	0.09	2500	62500	81768.38	5.24769	15581.78551
**20**	175	4	214	0.08	2500	62500	106844.99	18.7634	5694.329919
**21**	154	4	130	0.09	2500	62500	75229.51	4.89756	15360.61018
**22**	152	4	141	0.08	2500	62500	81264.47	7.97157	10194.2867
**23**	177	4	131	0.08	2500	62500	99403.59	4.8433	20523.93822
**24**	180	4	188	0.08	2500	62500	159339.42	14.8687	10716.43251

AFF GFAP-Tau quantification data

Case	Total Markers Counted	Number of Sections	Number of Sampling Sites	Coefficient of Error (Gundersen). m=1	Counting Frame Area (XY) (µm²)	Sampling Grid Area (XY) (µm²)	Grid Spacing (µm)	Area Sampling Fraction	Area Fraction
**AD cases**									
**1**	20366	4	100	0.0370	22500	810000	10	0.0278	0.0306
**2**	3339	4	67	0.041	22500	1000000	20	0.0225	0.0860
**3**	2252	4	44	0.045	22500	1000000	20	0.0225	0.0675
**4**	2358	4	48	0.0390	22500	1000000	20	0.0225	0.0679
**5**	2606	4	59	0.0680	22500	1000000	20	0.0225	0.0234

AFF GFAP- Aβ quantification data

Case	Total Markers Counted	Number of Sections	Number of Sampling Sites	Coefficient of Error (Gundersen). m=1	Counting Frame Area (XY) (µm²)	Sampling Grid Area (XY) (µm²)	Grid Spacing (µm)	Area Sampling Fraction	Area Fraction
**AD cases**									
**1**	20366	4	100	0.0750	22500	810000	10	0.0278	0.0016
**2**	17746	4	85	0.092	22500	810000	10	0.0278	0.0014
**3**	10818	4	58	0.054	22500	810000	10	0.0278	0.0047
**4**	10539	4	60	0.0840	22500	810000	10	0.0278	0.0027
**5**	16779	4	87	0.0850	22500	640000	10	0.0352	0.0015

AFF GFAP-Tau- Aβ quantification data

Case	Total Markers Counted	Number of Sections	Number of Sampling Sites	Coefficient of Error (Gundersen). m=1	Counting Frame Area (XY) (µm²)	Sampling Grid Area (XY) (µm²)	Grid Spacing (µm)	Area Sampling Fraction	Area Fraction
**AD cases**									
**1**	20366	4	100	0.0700	22500	810000	10	0.0278	0.0024
**2**	17746	4	85	0.096	22500	810000	10	0.0278	0.0013
**3**	34264	4	179	0.095	22500	250000	10	0.0900	0.0008
**4**	14331	4	71	0.0850	22500	640000	10	0.0352	0.0018
**5**	67435	4	346	0.092	22500	160000	10	0.1406	0.0004

AFF Tau quantification data

Case	Total Markers Counted	Number of Sections	Number of Sampling Sites	Coefficient of Error (Gundersen). m=1	Counting Frame Area (XY) (µm²)	Sampling Grid Area (XY) (µm²)	Grid Spacing (µm)	Area Sampling Fraction	Area Fraction
**AD cases**									
**1**	20140	4	100	0.0330	22500	810000	10	0.0278	0.0705
**2**	3339	4	67	0.043	22500	1000000	20	0.0225	0.1641
**3**	2252	4	44	0.041	22500	1000000	20	0.0225	0.1837
**4**	2358	4	48	0.019	22500	1000000	20	0.0225	0.2379
**5**	2610	4	59	0.047	22500	1000000	20	0.0225	0.0981

AFF Aβ quantification data

Case	Total Markers Counted	Number of Sections	Number of Sampling Sites	Coefficient of Error (Gundersen). m=1	Counting Frame Area (XY) (µm²)	Sampling Grid Area (XY) (µm²)	Grid Spacing (µm)	Area Sampling Fraction	Area Fraction
**AD cases**									
**1**	20140	4	100	0.0680	22500	810000	10	0.0278	0.0026
**2**	17746	4	85	0.06	22500	810000	10	0.0278	0.0033
**3**	10818	4	58	0.032	22500	810000	10	0.0278	0.0105
**4**	10539	4	60	0.071	22500	810000	10	0.0278	0.0042
**5**	16779	4	87	0.054	22500	640000	10	0.0352	0.0035

AFF Tau- Aβ quantification data

Case	Total Markers Counted	Number of Sections	Number of Sampling Sites	Coefficient of Error (Gundersen). m=1	Counting Frame Area (XY) (µm²)	Sampling Grid Area (XY) (µm²)	Grid Spacing (µm)	Area Sampling Fraction	Area Fraction
**AD cases**									
**1**	20140	4	100	0.0950	22500	810000	10	0.0278	0.0038
**2**	17746	4	85	0.096	22500	810000	10	0.0278	0.0013
**3**	34264	4	179	0.077	22500	250000	10	0.0900	0.0012
**4**	14331	4	71	0.056	22500	640000	10	0.0352	0.0031
**5**	67435	4	346	0.089	22500	160000	10	0.1406	0.0005
