# Supplementary material for: Neurodegeneration and Astrogliosis in the Human CA1 Hippocampal Subfield Are Related to hsp90ab1 and bag3 in Alzheimer’s Disease
Source: Int J Mol Sci. 2021 Dec 23;23(1):165. doi: 10.3390/ijms23010165 (PMC8745315; doi:10.3390/ijms23010165)
Supplement: Supplementary file 1 [file ijms-23-00165-s001.zip › File S2.pdf]

Detailed LC and mass spectrometry parameters.

Data-dependent acquisition method.

All samples were pooled and analysed by LC-MS/MS using a gas-phase fractionation approach (six methods, each one filtering the ions being analysed in the m/z range 349-450, 449-550, 549-650, 649-750, 749-900, 899-1200) in order to increase the number of identified peptides and proteins. Data was acquired in a hybrid Q-TOF mass spectrometer (Triple TOF 5600+, Sciex, Redwood City, CA, USA) coupled online to nano-HPLC (Ekspert nLC415, Eksigent, Dublin, CA, USA.).

A top 50 method was used, consisting on repeating cycles of a 250 ms TOF MS survey scan followed by a 50 ms MS/MS scan of the highest 50 precursor ions found in the survey scan. LC was performed at nano-flow (300 nL/min) in a 25 cm long x 75 µm internal diameter column (Acclaim PepMap 100, Thermo Scientific, Waltham, MA, USA) using a 90 min gradient from 5% to 25% B (A: 0.1% FA in water; B: 0.1% in ACN).

Proteins were identified using Protein Pilot software (v5.0.1, Sciex) with a human Swiss-Prot protein reference database (20416 protein entries, downloaded from UniProt on July 4th 2019). Protein identification settings used were: Iodoacetamide as Cys alkylation, trypsin as enzyme, TripleTOF 5600 as instrument and thorough ID as search effort. The false discovery rate (FDR) was set to 0.01 for both peptides and proteins.

SWATH method.

The method consisted on repeating a cycle of 50 TOF MS/MS scans of overlapping sequential precursor isolation windows of variable width (1 m/z overlap) covering the 350 to 1200 m/z mass range, with a previous MS scan for each cycle. The accumulation time was 50 ms for the MS scan (from 350 to 1200 m/z) and 90 ms for the product ion scan (230 to 1500 m/z, high sensitivity mode), thus making a 4.6 s total cycle time. The width of each of the 50 variable windows was optimized according to the ion density found in the previous DDA runs.
