# Supplementary material for: Neurodegeneration and Astrogliosis in the Human CA1 Hippocampal Subfield Are Related to hsp90ab1 and bag3 in Alzheimer’s Disease
Source: Int J Mol Sci. 2021 Dec 23;23(1):165. doi: 10.3390/ijms23010165 (PMC8745315; doi:10.3390/ijms23010165)
Supplement: Supplementary file 1 [file ijms-23-00165-s001.zip › Table S1.pdf]

Table S1. Antibodies detail.

Antigen	Antibody	Manufacturer	Cat. Nº	Dilution	Blocking buffer	Antibody buffer
**Immunohistochemistry**						
**NeuN**	Rabbit anti-NeuN	Abcam	ab104225	1:500	PBS- 0.4% Tx100- 10% NHS	PBS- 0.4% Tx100- 10% NHS
**Iba-1**	Rabbit anti-Iba-1	Wako	019-19741	1:2000	PBS- 0.1% Tx100	PBS- 0.1% Tx100
**GFAP**	Rabbit anti-GFAP	Dako	Z0334	1:10000	PBS- 0.1% Tx100- 10% NHS	PBS- 0.1% Tx100- 10% NHS
**Aβ**	Rabbit anti- β- amyloid	Cell Signaling	2454	1:250	PBS- 0.3% Tx100-2%NDS	PBS- 0.3% Tx100-2%NDS
**Aβ**	Mouse anti- β- amyloid	Merck	05-831-I	1:1000	TBS-0.1% Tx100-10% NDS	TBS-0.3% Tx100
**Tau**	Mouse anti- Tau	Cell Signaling	4019	1:800	PBS- 0.3% Tx100-2%NDS	PBS- 0.3% Tx100-2%NDS
**Tau**	Rabbit anti-Tau	Cell Signaling	46687	1:100	PBS- 0.3% Tx100	PBS- 0.3% Tx100
**GFAP**	Goat anti- GFAP	Abcam	ab53554	1:500	PBS- 0.3% Tx100-2%NDS	PBS- 0.3% Tx100-2%NDS
**BAG3**	Rabbit anti-BAG3	Invitrogen	PA5-53818	1:100	TBS-0.1% Tx100-10% NDS	TBS-0.3% Tx100
**HSP90AA1**	Mouse anti- HSP90α	Invitrogen	MA3-010	1:20	PBS- 0.3% Tx100	PBS- 0.3% Tx100
**HSP90AB1**	Mouse anti- HSP90β	Invitrogen	37-9400	1:20	PBS- 0.3% Tx100	PBS- 0.3% Tx100
**Western Blot**						
**GAPDH**	Rabbit anti-GAPDH	Cell Signaling	2118	1:10000	5% low-fat milk in TTBS	5% BSA in TTBS
**BAG3**	Rabbit anti-BAG3	Invitrogen	PA5-53818	1:1000	5% low-fat milk in TTBS	0.5% low-fat milk in TTBS
**HSP90AA1**	Mouse anti- HSP90α	Invitrogen	MA3-010	1:500	5% low-fat milk in TTBS	0.5% low-fat milk in TTBS
**HSP90AB1**	Mouse anti- HSP90β	Invitrogen	37-9400	1:1000	5% low-fat milk in TTBS	2.5% low-fat milk in TTBS
