# Supplementary figures and images for: Neurodegeneration and Astrogliosis in the Human CA1 Hippocampal Subfield Are Related to hsp90ab1 and bag3 in Alzheimer’s Disease
Source: Int J Mol Sci. 2021 Dec 23;23(1):165. doi: 10.3390/ijms23010165 (PMC8745315; doi:10.3390/ijms23010165)

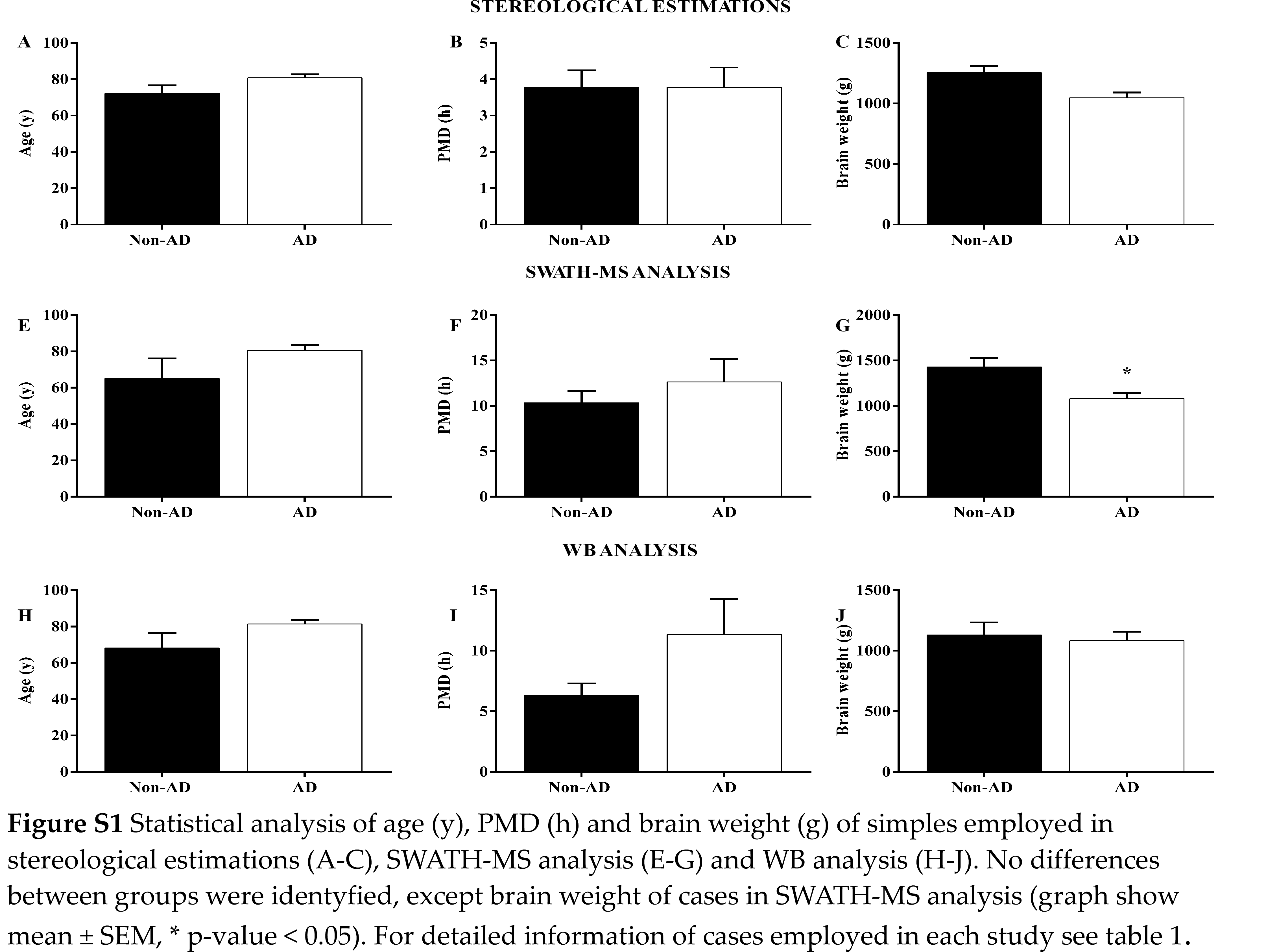

Supplement: Supplementary file 1 [file ijms-23-00165-s001.zip › Figure S1.jpg]
